# Supplementary material for: Cooperativity of ESPT and Aggregation-Induced Emission Effects—An Experimental and Theoretical Analysis of a 1,3,4-Thiadiazole Derivative
Source: Int J Mol Sci. 2024 Mar 15;25(6):3352. doi: 10.3390/ijms25063352 (PMC10970551; doi:10.3390/ijms25063352)
Supplement: Supplementary file 1 [file ijms-25-03352-s001.zip › ijms-2881139-supplementary.pdf]

# Supplementary materials

## Cooperativity of ESPT and Aggregation-Induced Emission Effects – an Experimental and Theoretical Analysis of a *1,3,4*-Thiadiazole Derivative

Iwona Budziak-Wieczorek[a], Dominika Kaczmarczyk[b], Klaudia Rząd[c], Mariusz Gagoś[d,e], Andrzej Stepulak[e], Beata Myśliwa-Kurdziel[f], Dariusz Karcz[g,i], Karolina Starzak[g], Gotard Burdziński[h], Monika Srebro-Hooper[*b], Arkadiusz Matwijczuk[*c,i]

**Correspondence**: arkadiusz.matwijczuk@up.lublin.pl (A.M.), tel.: +48-81-445-6909 (A.M.); monika.srebro@uj.edu.pl (M.S.-H.), tel.: +48-12-686-2383 (M.S.-H.)

## Experimental details

### Electronic absorption and fluorescence spectra

A double-beam UV-vis spectrophotometer Cary 300 Bio (Varian) equipped with a thermostatted tray holder with a 6×6 multi-cell Peltier block was used to record electronic absorption spectra for NTBD. A thermocouple probe (Cary Series II from Varian) was placed directly in the sample to monitor temperature.

Fluorescence excitation, emission and synchronous spectra were all measured at 22 ºC using a Cary Eclipse spectrofluorometer (Varian). The fluorescence spectra were recorded at 0.5 nm resolution with spectral characteristics corrections applied for the lamp and photomultiplier. Resonance light scattering (RLS) measurements were performed in line with the protocol previously reported in Ref. 1, with synchronous scanning of both the excitation and emission monochromators (with no interval between the excitation and emission wavelengths), at the spectral resolution of 1.5 nm. The recorded data was analyzed with Grams/AI 8.0 software (Thermo Electron Corporation; Waltham, Massachusetts, United States).

### Fluorescence quantum yields

Quantum yields (Φ) of fluorescence for NTBD were calculated relative to 7-diethylamino-4-methylcoumarin (coumarin1) taken as standard ($\Phi_F = 0.73$ in ethanol).[2] The final fluorescence quantum yield values were obtained from Equation (1):

$$\Phi_{F(X)} = \Phi_{R(EtOH)} \left(\frac{\lambda_{exR(EtOH)}}{\lambda_{exX}}\right)\left(\frac{I_X}{I_{R(EtOH)}}\right)\left(\frac{\eta_X^2}{\eta_{R(EtOH)}^2}\right) \quad (1)$$

where the subscript $X$ denotes NTBD in the solvent or mixed system and the subscript $R(EtOH)$ denotes coumarin1 in the EtOH solution; $\lambda_{ex}$ is the value of absorbance at the given excitation wavelength, $I$ is the area under the emission curve, and $\eta$ is the refractive index of the medium.

**Rate constants in radiative ($k_r$) and non-radiative ($k_{nr}$) intramolecular processes**

Quantum yield (Φ), defined as the numerical ratio of photons emitted to photons absorbed, is expressed in terms of radiative and non-radiative processes rate constants, $k_r$ and $k_{nr}$, as in Equation (2):[3]

$$\Phi = \frac{k_r}{k_{nr}+k_r} = \frac{number\ of\ photon\ emitted}{number\ of\ photon\ absorbed} \quad (2)$$

The value of fluorescence lifetime ($\tau$) relates to $k_r$ and $k_{nr}$ as shown in Equation (3):

$$\tau = \frac{1}{k_r+k_{nr}} \quad (3)$$

As such, the rate constants for radiative and non-radiative processes can be expressed in terms of the quantum yield and fluorescence lifetime in a given system, and calculated using Equations (4) and (5):[4]

$$k_r = \frac{\Phi}{\tau} \quad (4)$$

$$k_{nr} = \frac{1-\Phi}{\tau} \quad (5)$$

**Fluorescence lifetimes**

The frequency domain method was employed to measure fluorescence lifetimes ($\tau$) using a multifrequency cross-correlation phase and modulation K2 fluorometer (ISS, Champaign, IL). NTBD was dissolved in an organic solvent or a mixture of methanol and water. In each sample, the fluorescence emission was recorded in a 10×10 mm quartz cuvette (4×4 mm for high NTBD concentrations) with excitation at 315 nm (300 W xenon arc lamp), using a cut-off filter (transmittance for λ > 320 nm) in the emission channel. The measurements were carried out at 15-20 modulation frequencies within the range from 2 to 200 MHz, with a water solution of Ludox@ (Aldrich, Darmstad, Germany) used for reference. Data analyses were conducted with the Vinci 2.0 software from ISS, based on the multiexponential decay model for discrete fluorescence lifetime components shown in Equation (6):

$$I(l,t) = \sum_i \frac{f_i(l)}{t_i} e^{-t/t_i} \quad (6)$$

where $I(l,t)$ is the fluorescence intensity and $f_i(l)$ is the fractional contribution of each fluorescence lifetime component $t_i$.

Best-fit parameters were calculated by minimizing the reduced $\chi 2$ value and the residual distribution of the experimental data. Each measurement was taken 3-5 times after which the obtained results were averaged and standard deviations were calculated.

### Additional experimental data

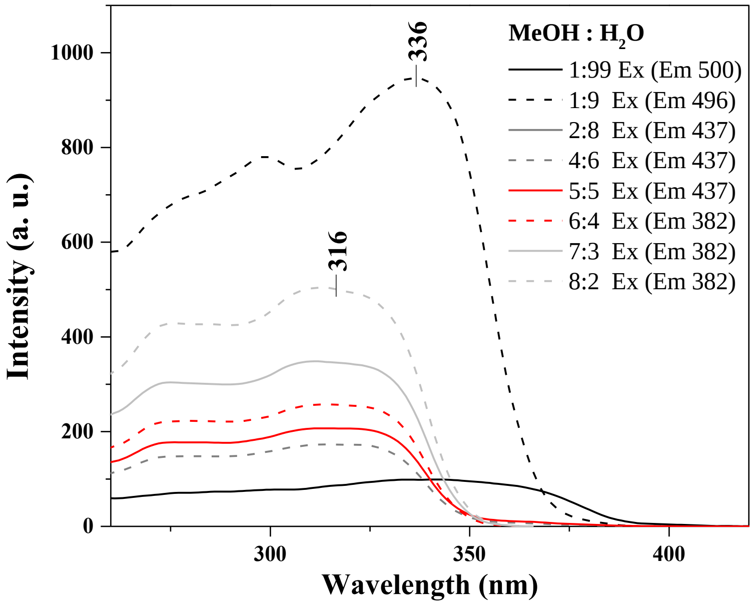


**Figure S1.** Fluorescence excitation spectra of NTBD in MeOH / $H_2O$ solutions with various volume ratio including 1:99, 1:9, 2:8, 4:6, 5:5, 6:4, 7:3 and 8:2. The excitation was set at the fluorescence emission maximum of each sample as noted in the legend.

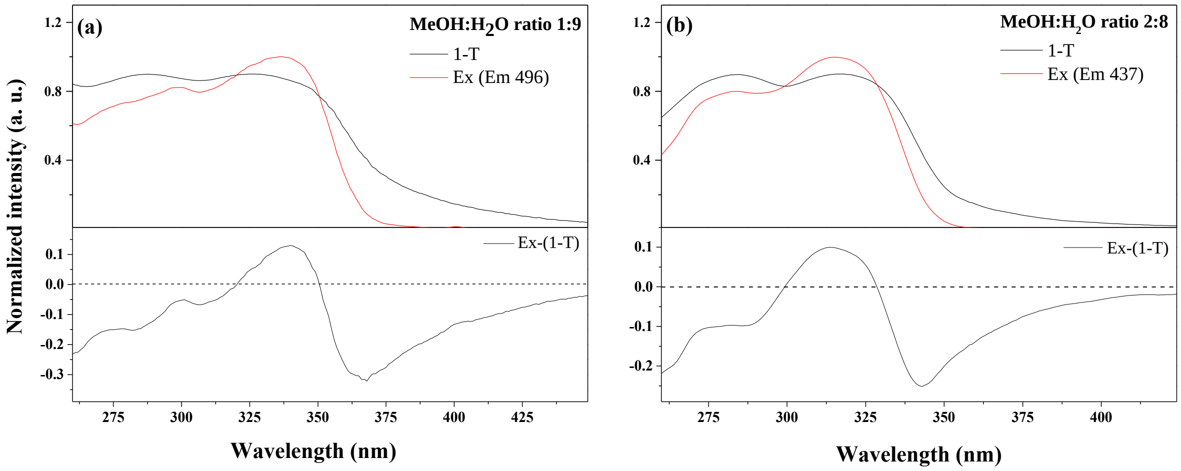


**Figure S2.** Top panels: Comparison of fluorescence excitation spectra (normalized to unity at the maximum intensity) and 1-T spectra (T: transmission) of NTBD dissolved in MeOH / $H_2O$ solutions with various volume ratio: 1:9 (panel a) and 2:8 (panel b). Bottom panels: The corresponding differential spectra Ex-(1-T).

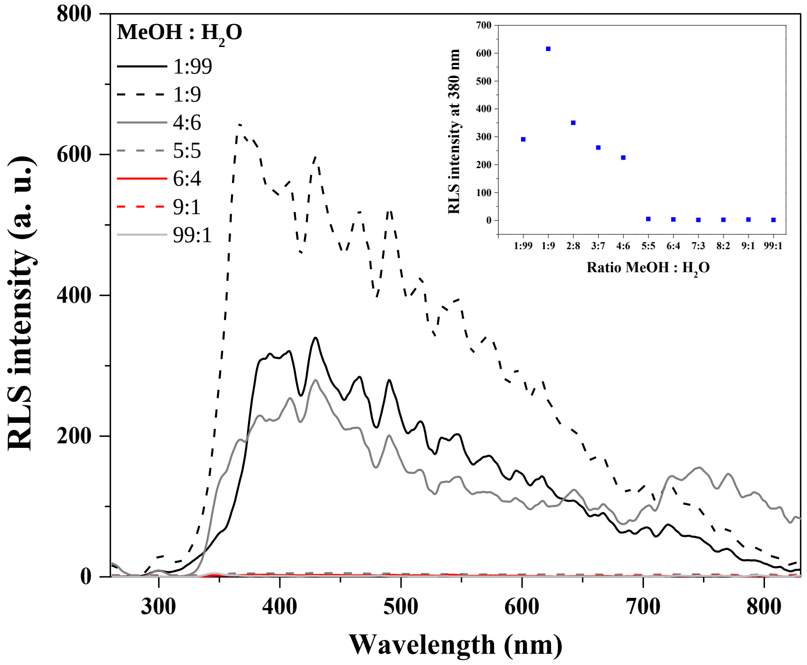


**Figure S3.** Resonance light scattering (RLS) spectra of NTBD in MeOH / $H_2O$ solutions with various volume ratio. The inserted graph presents RLS intensity at 380 nm as a function of the MeOH : $H_2O$ volume ratio of the NTBD sample.

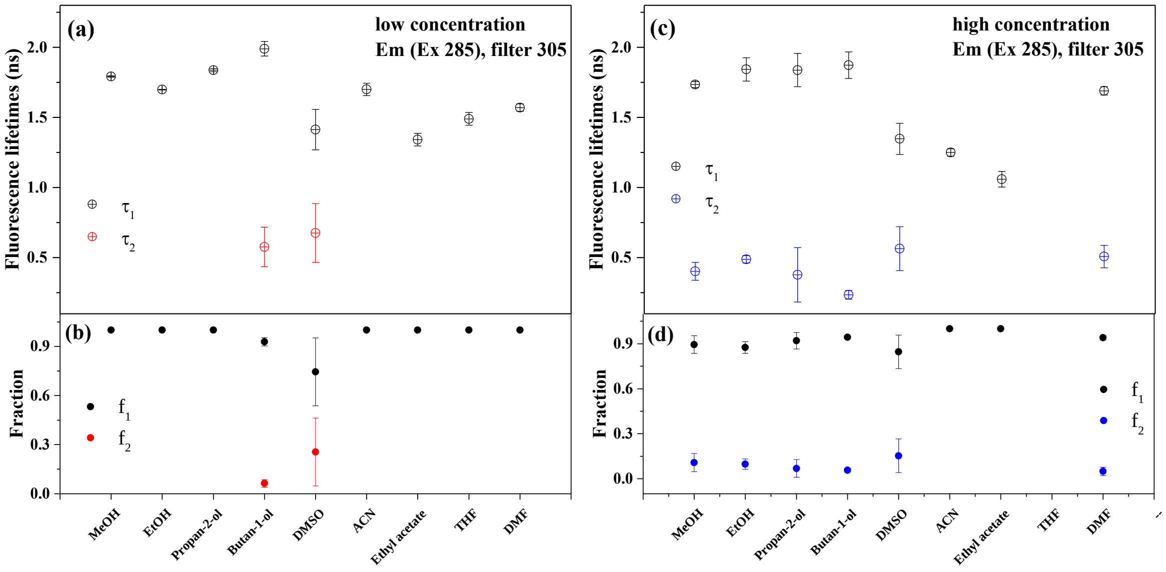


**Figure S4.** Fluorescence lifetimes (panels a and c) and fractional intensities (panels b and d), each with appropriate standard deviations indicated by bars, measured for NTBD in various solvents at low (left panels) and high (right panels) concentration employing 305 nm filter. Compare with Table S1.

**Table S1.** Fluorescence lifetimes (τ) and fractional intensities (f), along with their corresponding standard deviations, measured for NTBD in various solvents at low and high concentration. Measurements were performed with 305 nm filter at excitation wavelength of 285 nm. Compare with Figure S4.

| **Solvent/low concentration/ filter 305** | **$\tau_1$ ± sd** | **$f_1$ ± sd** | **$\tau_2$ ± sd** | **$f_2$ ± sd** |
|---|---|---|---|---|
| MeOH | 1.793 ± 0.005 | 1.000 | - | - |
| EtOH | 1.699 ± 0.003 | 1.000 | - | - |
| Propan-2-ol | 1.837 ± 0.011 | 1.000 | - | - |
| Butan-1-ol | 1.990 ± 0.052 | 0.928 ± 0.025 | 0.577 ± 0.142 | 0.063 ± 0.023 |
| DMSO | 1.413 ± 0.145 | 0.745 ± 0.207 | 0.676 ± 0.210 | 0.255 ± 0.207 |
| ACN | 1.700 ± 0.044 | 1.000 | - | - |
| Ethyl acetate | 1.342 ± 0.045 | 1.000 | - | - |
| THF | 1.490 ± 0.046 | 1.000 | - | - |
| DMF | 1.570 ± 0.026 | 1.000 | - | - |
| **Solvent/high concentration/ filter 305** | **$\tau_1$ ± sd** | **$f_1$ ± sd** | **$\tau_2$ ± sd** | **$f_2$ ± sd** |
| MeOH | 1.735 ± 0.021 | 0.895 ± 0.059 | 0.403 ± 0.064 | 0.107 ± 0.061 |
| EtOH | 1.843 ± 0.083 | 0.876 ± 0.039 | 0.488 ± 0.026 | 0.097 ± 0.035 |
| Propan-2-ol | 1.838 ± 0.119 | 0.920 ± 0.056 | 0.378 ± 0.195 | 0.069 ± 0.059 |
| Butan-1-ol | 1.873 ± 0.095 | 0.943 ± 0.012 | 0.236 ± 0.031 | 0.057 ± 0.012 |
| DMSO | 1.348 ± 0.111 | 0.846 ± 0.112 | 0.564 ± 0.157 | 0.153 ± 0.113 |
| ACN | 1.250 ± 0.026 | 1.000 | - | - |
| Ethyl acetate | 1.060 ± 0.056 | 1.000 | - | - |
| THF | - | - | - | - |
| DMF | 1.690 ± 0.030 | 0.940 ± 0.015 | 0.508 ± 0.080 | 0.049 ± 0.028 |

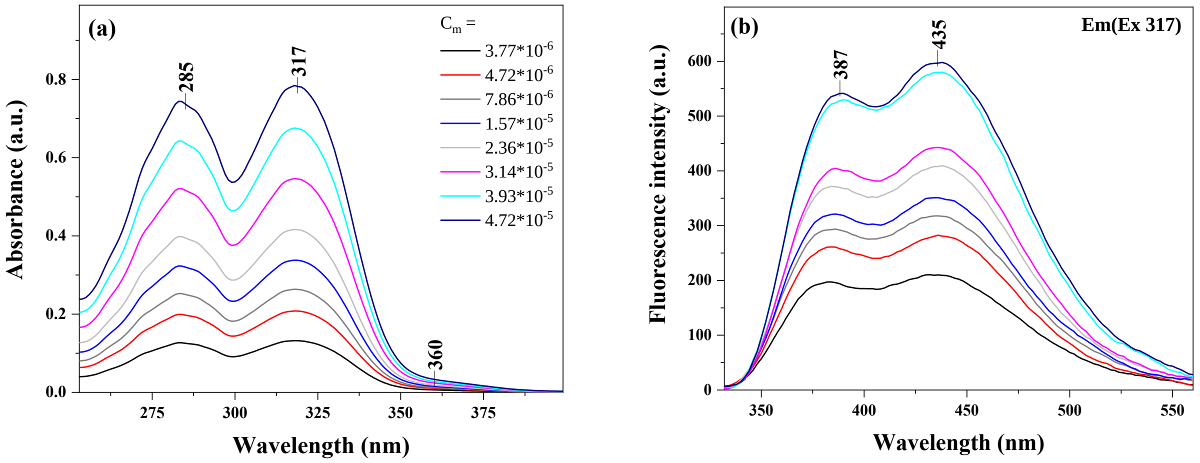


**Figure S5.** Electronic absorption spectra (panel a) and fluorescence emission spectra (panel b) measured at different concentrations of NTBD in MeOH / $H_2O$ solution with volume ratio of 5:5.

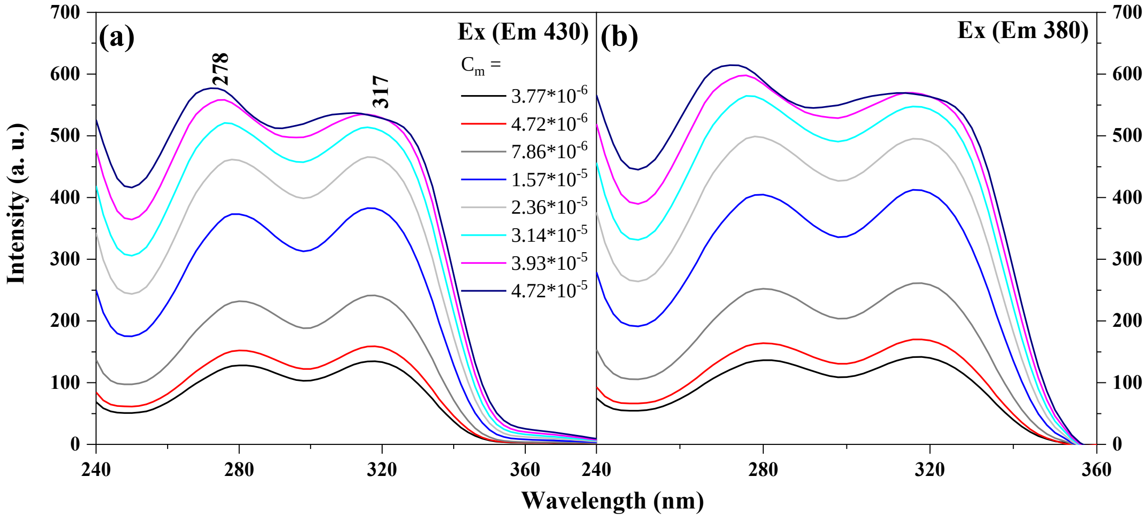


**Figure S6.** Fluorescence excitation spectra measured for the excitation at 430 nm (panel a) and 380 nm (panel b) at different concentrations of NTBD in MeOH / $H_2O$ solution with volume ratio of 5:5.

**Table S2.** Spectroscopic data for NTBD dissolved in different solvents: wavelengths of maximum absorbance and fluorescence emission (measured at excitation wavelength corresponding to a given maximum absorbance). Physical constants of solvents: dielectric constant ε, index of refraction n and solvent polarity functions $E_T(30)$ parameter.

| Solvent | Absorbance (nm) | Fluorescence (nm) | ε | n | $E_T(30)$ |
|---|---|---|---|---|---|
| **Methanol** | 321 | 373 | 33 | 1.3265 | 55.5 |
| | 286 | | | | |
| **Ethanol** | 322 | 374 | 25.3 | 1.3594 | 51.9 |
| | 286 | | | | |
| **Propan-2-ol** | 324 | 373 | 20.18 | 1.3772 | 48.6 |
| | 286 | | | | |
| **Butan-1-ol** | 325 | 375 | 17.8 | 1.3993 | 49.7 |
| | 286 | | | | |
| **Acetone** | 325 | 365 | 21 | 1.3587 | 42.2 |
| **DMSO** | 320 | 375 | 47.24 | 1.4773 | 45.1 |
| | 287 | | | | |
| **DMF** | 320 | 373 | 36.7 | 1.4305 | 43.2 |
| | 286 | | | | |
| **Acetonitrile** | 322 | 365 | 36.64 | 1.3416 | 45.6 |
| | 286 | 365/445 | | | |
| **Cyclohexane** | 330 | 490 | 2.02 | 1.4262 | 30.9 |
| | 285 | | | | |
| **Ethyl acetate** | 324 | 365 | 6.02 | 1.3723 | 38.1 |
| | 288 | 359 | | | |
| **THF** | 325 | 364 | 7.6 | 1.404 | 37.4 |
| | 290 | | | | |
| **Toluene** | 326 | 504 | 2.38 | 1.4969 | 33.9 |
| | 290 | 405 | | | |
| **Chloroform** | 326 | 370/505 | 4.81 | 1.4429 | 39.1 |
| | 286 | 370/505 | | | |
| **n-Hexane** | 320 | 490 | 1.89 | 1.3723 | 30.9 |
| | 280 | 410/490 | | | |
| **n-Heptane** | 326 | 490 | 1.92 | 1.3876 | 31.1 |
| | 286 | | | | |

## Computational details

All calculations were performed with density functional theory (DFT) and its time-dependent variant (TDDFT) methods using the Gaussian 16 package, version C.01.[5] Solvent effects for methanol (ε = 32.613) and water (ε = 78.3553) were modelled using the polarizable continuum model (PCM),[6,7,8,9,10] with default parameters of the Gaussian/PCM implementation. Symmetry was not explicitly imposed in any computations. All (unconstrained) geometry optimizations (for both $S_0$ and $S_1$) were followed by frequencies calculations at the same level of theory in order to confirm that obtained structures represent energy minima (no imaginary frequencies) and also to evaluate free energy values.

Systematic conformational analysis of NTBD in its two tautomeric forms – that is, enol and keto, with the former considered in two conformations: *cis* with the hydrogen atom of *ortho* OH group in the resorcynyl fragment directed towards the 1,3,4-thiadiazole ring and *trans* in which this atom is positioned away from the 1,3,4-thiadiazole – was carried out using the global hybrid B3LYP[11,12,13] exchange-correlation functional and split-valence triple-zeta basis set with one set of polarization functions and one set of diffuse functions for all atoms, 6-311++G(d,p),[14,15] and involved rotations around several bonds as indicated in Figure S7. Dispersion effects were accounted for in these calculations via the third-generation Grimme's set of semiempirical dispersion corrections with the Becke-Johnson damping, D3.[16,17] Optimized low-energy geometries for all these three structures – *cis*-enol, *trans*-enol, and keto – are presented in Figure S8 and characterized in Table S3. Note that the corresponding structures differing only in the position of the hydrogen atom of *para* OH group in the resorcynyl fragment are referred to as labelled without and with the prime symbol.

The absorption spectra and emission properties via $S_1$ excited-state geometry optimizations were computed with B3LYP and the aug-cc-pVDZ[18,19] basis set. The UV-vis spectra were simulated, based on the calculated vertical excitations from the ground state to the 100 lowest singlet excited states, by means of the GaussView 5.0.9 program[20] as a sum of Gaussian functions (with the broadening parameter set to 0.15 eV) centered at the excitation energies and scaled using the corresponding oscillator strengths. The character of the intense electronic excitations was examined by analyzing molecular orbitals (MOs) involved in dominant electronic transitions. MOs were visualized using the GaussView 5.0.9 program. Due to energetic and structural similarities of the (ground-state) structures differing only in the position of the hydrogen atom of *para* OH group in the resorcynyl fragment (labelled without and with the prime symbol), and more importantly high similarity of their corresponding simulated UV-

vis spectra (see Figure S9) and their electronic assignment (see Table S4 and Figure S10), $S_1$ excited-state geometry optimizations were performed only for the structures labelled without the prime symbol.

Energy barriers for the enol↔keto tautomerization in the $S_1$ excited state, that is for the excited-state intramolecular proton transfer (ESIPT) process, were determined based on the corresponding energy profile calculations for the proton transfer from the *cis*-enol structure to the respective keto at the B3LYP/aug-cc-pVDZ level. These calculations were carried out as a series of constrained (TDDFT) geometry optimizations with a frozen length of the N···H bonding in 0.1 Å increment (0.025 Å in the proximity of the TS region). For a comparison, the analogous (although DFT) calculations were performed in the $S_0$ ground state, following the re-optimization of the appropriate *cis*-enol and keto structures with B3LYP/aug-cc-pVDZ.

Finally, possibility of intramolecular rotation of the resorcynyl fragment relative to the 1,3,4-thiadiazole unit, that is around the $C_2$-$C_3$ bond (as defined in Figure S11), in the $S_1$ excited state (representing twisted intramolecular charge-transfer (TICT) effect) was examined for both the *cis*-enol and keto isomers based on the corresponding energy profile calculations at the TDDFT B3LYP/aug-cc-pVDZ level. These computations were performed as a series of constrained geometry optimizations with the frozen dihedral $C_1$-$C_2$-$C_3$-N angle (Figure S11) in 30 degree increment (7.5 degree in the proximity of selected TS regions).

The global hybrid B3LYP density functional was chosen for these studies due to its successful employment in quantum-chemical characterization of numerous 1,3,4-thiadiazole derivatives including resorcynyl-based ones.[21,22,23,24,25,26,27] While the correlation consistent polarized valence double-zeta diffuse-augmented (aug-cc-pVDZ) basis set was indicated in the aforementioned previous research[23,24,25] as providing a satisfactory agreement between the computed and experimental photophysical properties, for this project we initially chose a triple-zeta basis set with polarization and diffuse functions added, 6-311++G(d,p), of presumably better quality in terms of a description of valence electrons. Unfortunately, after the successful employment of B3LYP/6-311++G(d,p) for $S_0$ conformational analysis, the preliminary TDDFT excited state $S_1$ geometry optimizations showed that with such method no stable planar keto $S_1$ structure can be found as all the calculations led to the twisted geometry instead. Consequently, photophysical properties were finally computed with the aug-cc-pVDZ basis set that demonstrated similar results to 6-311++G(d,p) for enol $S_1$ but enabled to locate both planar and twisted keto $S_1$ forms.

## Additional computational data

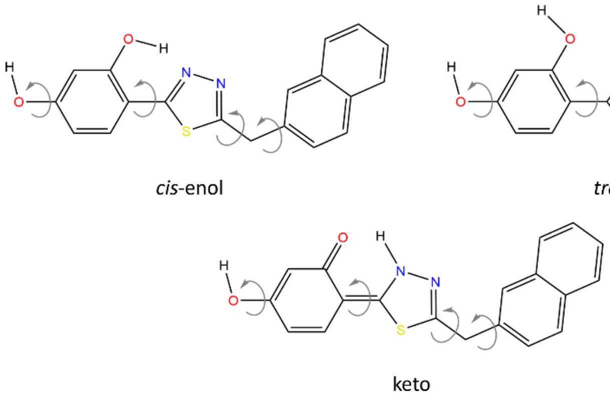


**Figure S7.** Schematic representation of the molecular structure of NTBD in its enolic (*cis* and *trans*) and keto forms subjected to a systematic conformational analysis employing dihedral angles indicated by curved arrows.

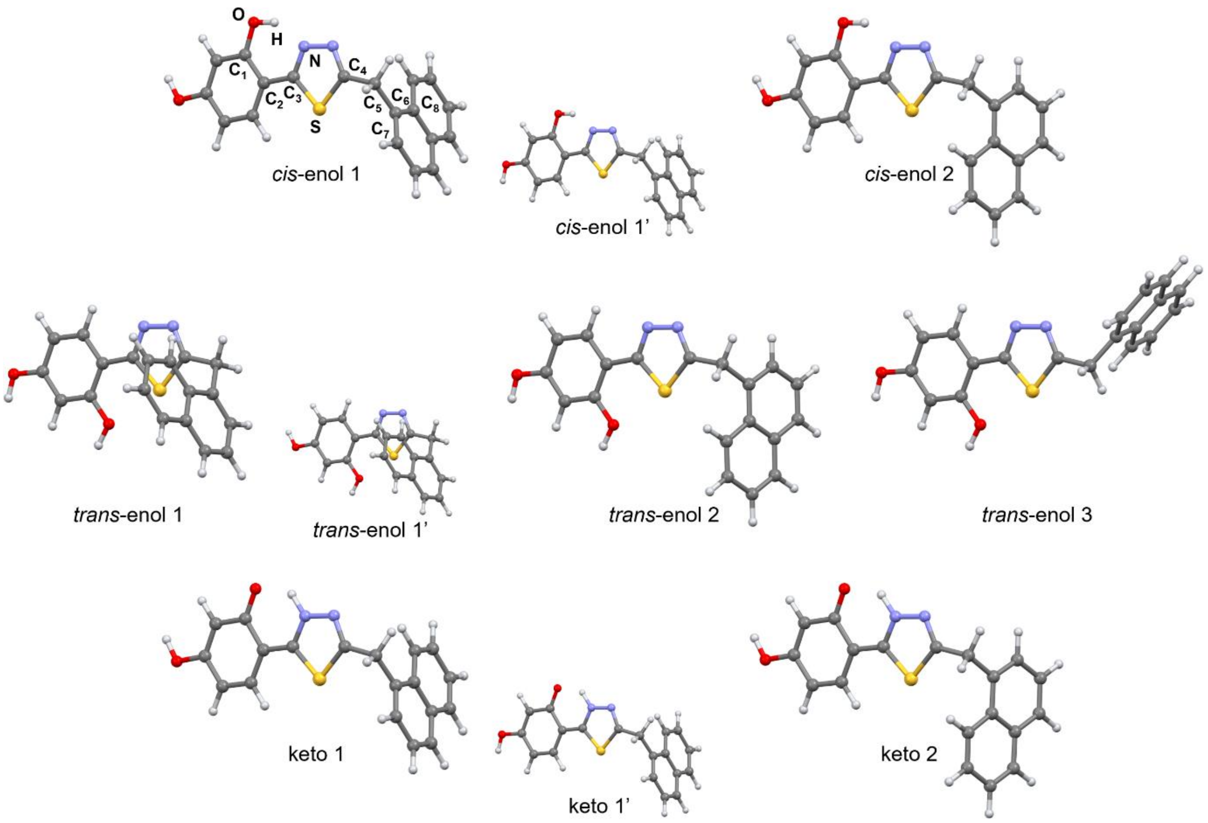


**Figure S8.** Selected DFT-optimized (B3LYP+D3/6-311++G(d,p) with continuum solvent model for MeOH) low-energy geometries of enolic (*cis* and *trans*) and keto forms of NTBD. See Table S3 for quantitative characterization of the presented structures.

**Table S3.** Relative energy and relative free energy values (respectively, Δ*E* and Δ*G*, in kcal/mol, computed with respect to the lowest-energy (*cis*-enol) structure) along with the corresponding selected structural parameters (dihedral angles ∠, in deg, and interatomic distances *d*, in Å) for different low-energy geometries of enolic (*cis* and *trans*) and keto forms of NTBD. For structures visualization and atoms labelling, see Figure S8. Note that the corresponding structures labelled without and with the prime symbol differ only in the position of 4-hydroxy group. Calculations were performed at DFT B3LYP+D3/6-311++G(d,p)/PCM(MeOH or $H_2O$) level; for a comparison, the corresponding B3LYP/aug-cc-pVDZ/PCM(MeOH or $H_2O$) results are also listed.

| Structure | Δ*E* | Δ*G* | $\angle C_1C_2C_3S$ | $\angle C_4C_5C_6C_7$ | $\angle C_4C_5C_6C_8$ | $d_{O\text{-}H}$ | $d_{N\text{-}H}$ |
|---|---|---|---|---|---|---|---|
| **B3LYP+D3/6-311++G(d,p)/PCM(MeOH)** | | | | | | | |
| ***cis*-enol 1** | 0.00 | 0.05 | -178.2 | -111.3 | 67.2 | 0.992 | 1.726 |
| ***cis*-enol 1'** | 0.07 | 0.00 | -178.9 | -111.0 | 67.4 | 0.992 | 1.728 |
| ***cis*-enol 2** | 1.11 | 0.75 | -179.6 | 89.4 | -88.4 | 0.992 | 1.726 |
| ***cis*-enol 2'** | 1.17 | 0.96 | -179.7 | 89.3 | -88.6 | 0.992 | 1.727 |
| ***trans*-enol 1** | 6.25 | 5.72 | -1.0 | 111.2 | -67.4 | 0.965 | – |
| ***trans*-enol 1'** | 6.06 | 5.65 | -0.7 | 111.1 | -67.5 | 0.965 | – |
| ***trans*-enol 2** | 7.30 | 6.92 | 0.6 | 89.7 | -88.2 | 0.965 | – |
| ***trans*-enol 2'** | 7.11 | 6.46 | 0.6 | 90.0 | -87.9 | 0.965 | – |
| ***trans*-enol 3** | 7.90 | 6.80 | -1.3 | 110.3 | -70.0 | 0.965 | – |
| ***trans*-enol 3'** | 7.70 | 6.72 | 0.8 | 110.2 | -70.1 | 0.965 | – |
| **keto 1** | 6.45 | 6.31 | -178.6 | -110.3 | 67.9 | 1.728 | 1.038 |
| **keto 1'** | 6.91 | 6.67 | -178.9 | -110.3 | 67.9 | 1.715 | 1.040 |
| **keto 2** | 7.58 | 7.17 | -179.6 | 89.4 | -88.2 | 1.726 | 1.038 |
| **keto 2'** | 8.06 | 7.62 | -179.6 | 89.3 | -88.4 | 1.713 | 1.040 |
| **B3LYP/aug-cc-pVDZ/PCM(MeOH)** | | | | | | | |
| ***cis*-enol 1** | 0.00 | 0.00 | -179.8 | -106.8 | 73.3 | 0.994 | 1.724 |
| ***cis*-enol 2** | 0.90 | 1.02 | 179.7 | 87.6 | -92.0 | 0.994 | 1.724 |
| **keto 1** | 6.87 | 6.48 | -179.8 | -106.3 | 73.7 | 1.678 | 1.048 |
| **keto 2** | 7.81 | 7.54 | 179.9 | 87.7 | -91.9 | 1.677 | 1.048 |
| **B3LYP+D3/6-311++G(d,p)/PCM($H_2O$)** | | | | | | | |
| ***cis*-enol 1** | 0.00 | 0.03 | -178.2 | -111.3 | 67.2 | 0.992 | 1.726 |
| ***cis*-enol 1'** | 0.05 | 0.00 | -179.2 | -111.2 | 67.4 | 0.992 | 1.727 |
| ***cis*-enol 2** | 1.10 | 0.71 | -179.6 | 89.4 | -88.4 | 0.992 | 1.726 |
| ***cis*-enol 2'** | 1.15 | 1.01 | -179.8 | 89.3 | -88.5 | 0.992 | 1.726 |

| | | | | | | | |
|---|---|---|---|---|---|---|---|
| ***trans*-enol 1** | 6.12 | 5.64 | -0.8 | 111.2 | -67.4 | 0.965 | – |
| ***trans*-enol 1'** | 5.96 | 5.56 | -0.6 | 111.1 | -67.5 | 0.965 | – |
| ***trans*-enol 2** | 7.16 | 6.74 | 0.4 | 89.8 | -88.1 | 0.965 | – |
| ***trans*-enol 2'** | 7.00 | 6.43 | 0.7 | 90.0 | -87.8 | 0.965 | – |
| ***trans*-enol 3** | 7.67 | 6.71 | -1.1 | 110.2 | -70.1 | 0.965 | – |
| ***trans*-enol 3'** | 7.51 | 6.61 | 1.3 | 110.1 | -70.1 | 0.965 | – |
| **keto 1** | 6.38 | 6.23 | -178.6 | -110.2 | 68.0 | 1.729 | 1.038 |
| **keto 1'** | 6.79 | 6.57 | -178.9 | -110.2 | 68.0 | 1.717 | 1.040 |
| **keto 2** | 7.50 | 7.10 | -179.6 | 89.4 | -88.2 | 1.728 | 1.038 |
| **keto 2'** | 7.94 | 7.53 | -179.6 | 89.3 | -88.4 | 1.715 | 1.040 |
| **B3LYP/aug-cc-pVDZ/PCM($H_2O$)** | | | | | | | |
| ***cis*-enol 1** | 0.00 | 0.00 | -179.9 | -107.0 | 73.2 | 0.994 | 1.724 |
| ***cis*-enol 2** | 0.90 | 0.89 | 179.7 | 87.6 | -92.0 | 0.994 | 1.724 |
| **keto 1** | 6.80 | 6.35 | -179.8 | -106.3 | 73.7 | 1.680 | 1.048 |
| **keto 2** | 7.74 | 7.37 | 179.9 | 87.6 | -91.9 | 1.679 | 1.048 |

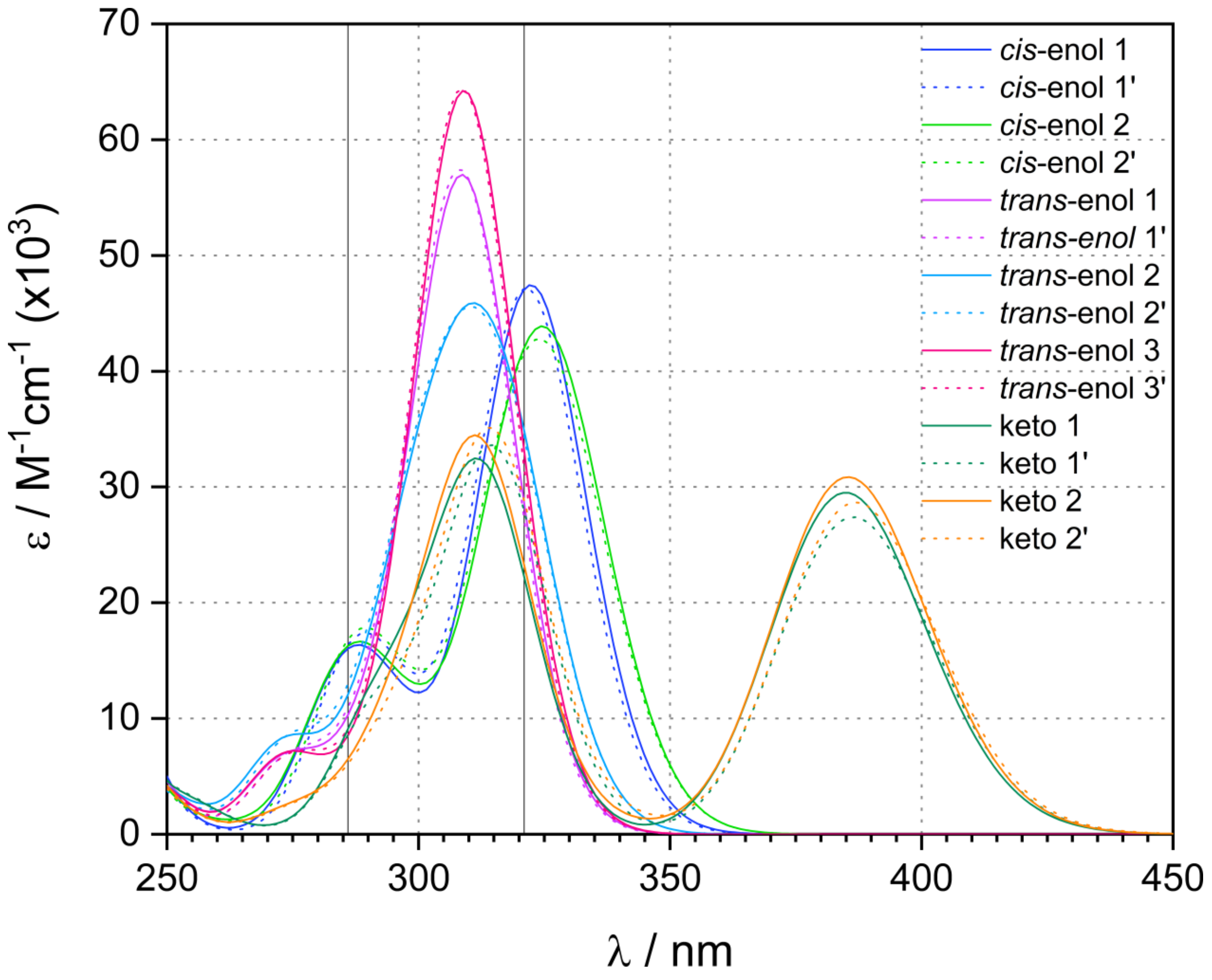


**Figure S9.** Simulated UV-vis spectra for different structures of enolic (*cis* and *trans*) and keto forms of NTBD. No spectral shift has been applied. Black vertical lines mark positions of maxima of two main bands observed in the experimental absorption spectrum of NTBD measured in MeOH. For structures visualization, see Figure S8; for selected dominant excitations and their MO-pair contributions, see Table S4; for MOs isosurfaces visualization, see Figure S10. Calculations were performed at TDDFT B3LYP/aug-cc-pVDZ/PCM(MeOH) level at the corresponding geometries optimized with DFT B3LYP+D3/6-311++G(d,p)/PCM(MeOH).

**Table S4.** Selected dominant excitations and occupied (occ) – unoccupied (unocc) MO-pair contributions (greater than 10%) for different structures of enolic (*cis* and *trans*) and keto forms of NTBD. For structures visualization, see Figure S8; for MOs isosurfaces visualization, see Figure S10; for the corresponding simulated UV-vis absorption spectra, see Figure S9. Calculations were performed at TDDFT B3LYP/aug-cc-pVDZ/PCM(MeOH) level at the

corresponding geometries optimized with DFT B3LYP+D3/6-311++G(d,p)/PCM(MeOH). MO number referring to HOMO and LUMO is indicated by (H) and (L), respectively.

| Structure | Excitation | *E* / eV | *λ* / nm | *f* | occ no. | unocc no. | % |
|---|---|---|---|---|---|---|---|
| ***cis*-enol 1** | 1 | 3.84 | 323 | 0.303 | 87(H) | 88(L) | 61.7 |
| | | | | | 86 | 88 | 34.2 |
| | 2 | 3.85 | 322 | 0.224 | 86 | 88 | 64.7 |
| | | | | | 87 | 88 | 32.1 |
| | 4 | 4.23 | 293 | 0.096 | 86 | 89 | 91.3 |
| | 5 | 4.37 | 284 | 0.113 | 85 | 88 | 83.2 |
| ***cis*-enol 1'** | 2 | 3.86 | 321 | 0.486 | 87(H) | 88(L) | 75.8 |
| | | | | | 86 | 88 | 17.7 |
| | 4 | 4.23 | 293 | 0.088 | 86 | 89 | 88.9 |
| | 5 | 4.34 | 286 | 0.114 | 85 | 88 | 82.8 |
| ***cis*-enol 2** | 1 | 3.77 | 329 | 0.313 | 87(H) | 88(L) | 95.7 |
| | 2 | 3.89 | 319 | 0.226 | 86 | 88 | 93.3 |
| | 4 | 4.24 | 292 | 0.060 | 86 | 89 | 93.6 |
| | 5 | 4.36 | 284 | 0.116 | 85 | 88 | 81.9 |
| ***cis*-enol 2’** | 1 | 3.77 | 329 | 0.285 | 87(H) | 88(L) | 97.0 |
| | 2 | 3.89 | 318 | 0.250 | 86 | 88 | 93.4 |
| | 3 | 4.18 | 297 | 0.048 | 87 | 89 | 91.5 |
| | 4 | 4.26 | 291 | 0.048 | 86 | 89 | 92.9 |
| | 5 | 4.33 | 286 | 0.122 | 85 | 88 | 82.2 |
| ***trans*-enol 1** | 1 | 4.00 | 310 | 0.221 | 87(H) | 88(L) | 48.6 |
| | | | | | 86 | 88 | 45.9 |
| | 2 | 4.02 | 309 | 0.393 | 86 | 88 | 52.0 |
| | | | | | 87 | 88 | 41.7 |
| | 4 | 4.24 | 293 | 0.090 | 86 | 89 | 90.1 |
| | 6 | 4.53 | 274 | 0.072 | 85 | 88 | 46.7 |
| | | | | | 87 | 91 | 21.9 |
| | | | | | 84 | 88 | 18.3 |
| ***trans*-enol 1’** | 1 | 4.00 | 310 | 0.094 | 86 | 88(L) | 63.3 |
| | | | | | 87(H) | 88 | 32.9 |
| | 2 | 4.02 | 309 | 0.523 | 87 | 89 | 97.8 |
| | 4 | 4.24 | 293 | 0.088 | 86 | 89 | 90.1 |
| | 6 | 4.51 | 275 | 0.071 | 85 | 88 | 66.5 |
| | | | | | 87 | 91 | 19.3 |

| | | | | | | | |
|---|---|---|---|---|---|---|---|
| ***trans*-enol 2** | 1 | 3.91 | 317 | 0.348 | 87(H) | 88(L) | 95.6 |
| | 2 | 4.07 | 305 | 0.218 | 86 | 88 | 84.6 |
| | 3 | 4.16 | 298 | 0.085 | 87 | 89 | 89.5 |
| | 4 | 4.25 | 292 | 0.061 | 86 | 89 | 89.2 |
| | 6 | 4.54 | 273 | 0.086 | 85 | 88 | 56.4 |
| | | | | | 87 | 91 | 15.6 |
| ***trans*-enol 2'** | 1 | 3.91 | 317 | 0.335 | 87(H) | 88(L) | 96.1 |
| | 2 | 4.07 | 305 | 0.235 | 86 | 88 | 84.7 |
| | 3 | 4.17 | 297 | 0.086 | 87 | 89 | 88.7 |
| | 6 | 4.52 | 274 | 0.086 | 85 | 88 | 62.7 |
| | | | | | 87 | 91 | 13.0 |
| ***trans*-enol 3** | 2 | 4.01 | 309 | 0.693 | 86 | 88(L) | 80.5 |
| | | | | | 87(H) | 88 | 11.1 |
| | 4 | 4.23 | 293 | 0.053 | 86 | 89 | 50.0 |
| | | | | | 87 | 89 | 41.6 |
| | 6 | 4.53 | 274 | 0.074 | 84 | 88 | 53.8 |
| | | | | | 86 | 91 | 18.6 |
| | | | | | 85 | 88 | 12.8 |
| ***trans*-enol 3'** | 2 | 4.01 | 309 | 0.700 | 86 | 88(L) | 90.4 |
| | 3 | 4.23 | 293 | 0.040 | 86 | 89 | 54.3 |
| | | | | | 87(H) | 89 | 38.6 |
| | 6 | 4.51 | 275 | 0.072 | 84 | 88 | 42.0 |
| | | | | | 85 | 88 | 27.2 |
| | | | | | 86 | 91 | 20.8 |
| **keto 1** | 1 | 3.22 | 385 | 0.328 | 87(H) | 88(L) | 97.2 |
| | 5 | 3.97 | 312 | 0.346 | 85 | 88 | 93.0 |
| | 6 | 4.23 | 293 | 0.099 | 86 | 89 | 91.6 |
| **keto 1'** | 1 | 3.21 | 386 | 0.305 | 87(H) | 88(L) | 97.2 |
| | 5 | 3.94 | 315 | 0.366 | 85 | 88 | 93.5 |
| | 6 | 4.23 | 293 | 0.100 | 86 | 89 | 92.7 |
| **keto 2** | 1 | 3.22 | 385 | 0.343 | 87(H) | 88(L) | 97.2 |
| | 5 | 3.97 | 312 | 0.359 | 85 | 88 | 92.2 |
| | 6 | 4.19 | 296 | 0.096 | 86 | 89 | 91.1 |
| **keto 2'** | 1 | 3.20 | 387 | 0.319 | 87(H) | 88(L) | 97.3 |
| | 5 | 3.94 | 315 | 0.375 | 85 | 88 | 92.5 |
| | 6 | 4.19 | 296 | 0.097 | 86 | 89 | 91.6 |

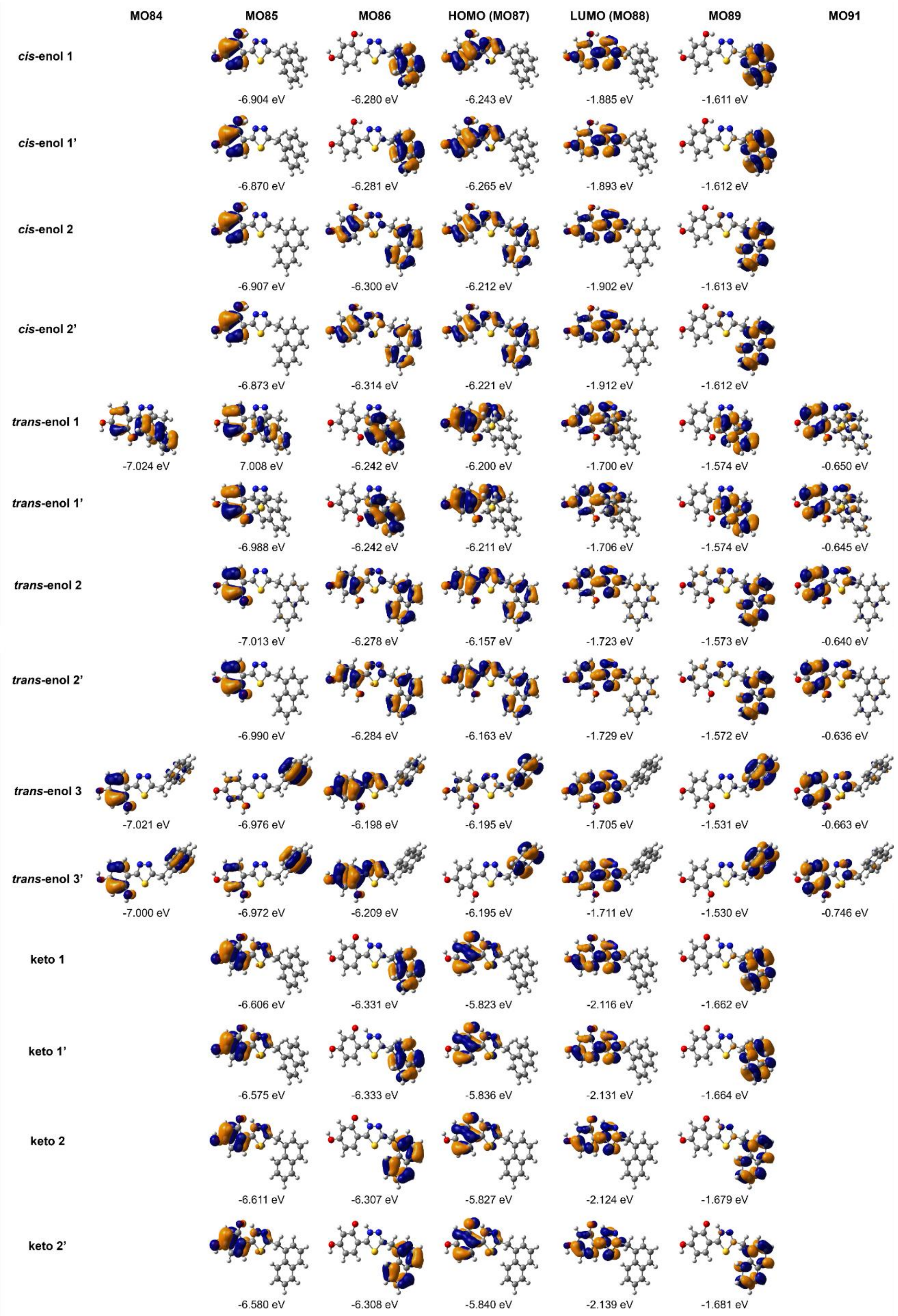


**Figure S10.** Isosurfaces (±0.04 au) of MOs involved in selected electronic transitions for different structures of enolic (*cis* and *trans*) and keto forms of NTBD, obtained based on DFT B3LYP/aug-cc-pVDZ/PCM(MeOH) calculations. Numbers listed are orbital energy values.

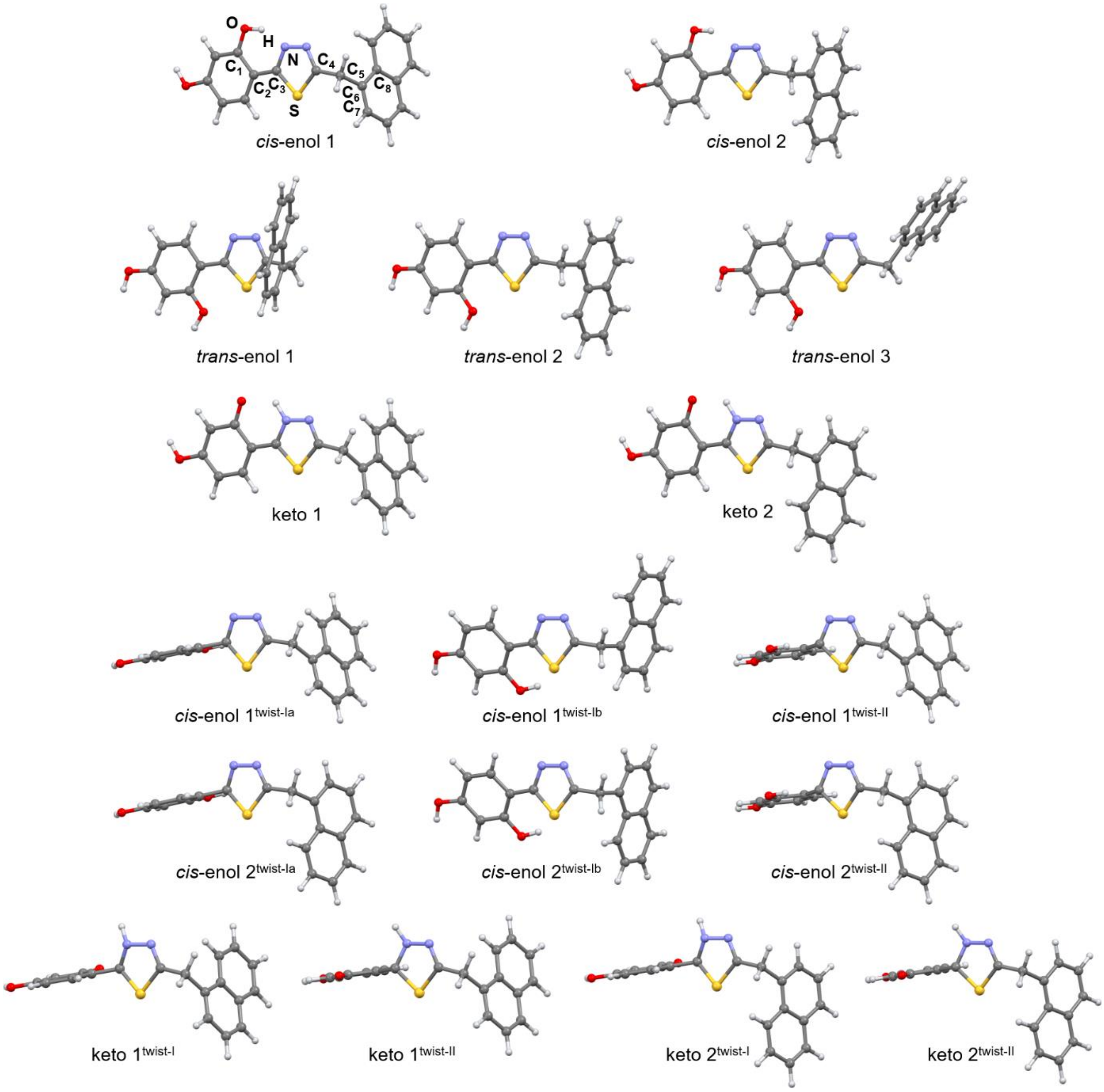


**Figure S11.** TDDFT-optimized (B3LYP/aug-cc-pVDZ with continuum solvent model for MeOH) $S_1$ excited-state structures of NTBD in its enolic (*cis* and *trans*) and keto forms, including both planar (top) and twisted (bottom) conformations. See Table S5 for quantitative characterization of the presented structures.

**Table S5.** Relative energy and relative free energy values (respectively, Δ*E* and Δ*G*, in kcal/mol, with respect to the lowest-energy planar (emissive) keto structure) and the corresponding selected structural parameters (dihedral angles ∠, in deg, and interatomic distances *d*, in Å) for different geometries of enolic (*cis* and *trans*) and keto forms of NTBD in its $S_1$ excited state along with the respective $S_1$-$S_0$ energy difference representing fluorescence energy $\lambda$, in nm, its corresponding oscillator strength *f*, and percentage contribution of LUMO→HOMO transition to the $S_1 \rightarrow S_0$ emission % L→H. For structures visualization and atoms labelling, see Figure S11. For MOs isosurfaces visualization, see Figure S12. Calculations were performed at TDDFT B3LYP/aug-cc-pVDZ/PCM(MeOH or $H_2O$) level.

| Structure | Δ*E* | Δ*G* | $\angle C_1C_2C_3S$ | $\angle C_4C_5C_6C_7$ | $\angle C_4C_5C_6C_8$ | $d_{O\text{-}H}$ | $d_{N\text{-}H}$ | $\lambda$ | *f* | % L→H |
|---|---|---|---|---|---|---|---|---|---|---|
| **MeOH** | | | | | | | | | | |
| ***cis*-enol 1** | 6.86 | 5.93 | 179.6 | -101.3 | 77.7 | 1.010 | 1.665 | 382 | 0.790 | 98 |
| ***cis*-enol 1$^{twist\text{-}Ia}$** | 16.84 | 15.95 | -114.5 | -107.4 | 72.8 | 1.014 | 2.795 | 510 | 0.001 | 99 |
| ***cis*-enol 1$^{twist\text{-}Ib}$** | 16.48 | 14.80 | -22.5 | -86.5 | 91.1 | 0.974 | 3.812 | 395 | 0.637 | 99 |
| ***cis*-enol 1$^{twist\text{-}II}$** | 16.72 | 16.24 | 117.9 | -107.7 | 72.3 | 1.014 | 2.760 | 513 | 0.004 | 99 |
| ***cis*-enol 2** | 5.97 | 5.81 | 177.7 | 88.6 | -87.9 | 1.003 | 1.688 | 397 | 0.684 | 99 |
| ***cis*-enol 2$^{twist\text{-}Ia}$** | 17.69 | 16.98 | -113.1 | 87.6 | -92.0 | 1.014 | 2.812 | 508 | 0.000 | 98 |
| ***cis*-enol 2$^{twist\text{-}Ib}$** | 15.81 | 14.74 | -24.3 | 88.2 | -88.4 | 0.973 | 3.807 | 398 | 0.633 | 99 |
| ***cis*-enol 2$^{twist\text{-}II}$** | 17.60 | 16.78 | 117.7 | 87.2 | -92.2 | 1.013 | 2.764 | 508 | 0.006 | 97 |
| ***trans*-enol 1** | 14.80 | 13.77 | 0.9 | 85.3 | -92.6 | 0.968 | – | 389 | 0.781 | 99 |
| ***trans*-enol 2** | 14.13 | 13.35 | -3.1 | 89.2 | -87.6 | 0.968 | – | 393 | 0.748 | 99 |
| ***trans*-enol 3** | 16.11 | 16.96 | 0.1 | 104.0 | -77.1 | 0.969 | – | 379 | 0.925 | 99 |
| **keto 1** | 0.00 | 0.68 | 178.1 | -107.0 | 72.9 | 1.953 | 1.022 | 469 | 0.338 | 99 |
| **keto 1$^{twist\text{-}I}$** | -6.84 | -5.75 | -111.9 | -107.2 | 73.0 | 3.754 | 1.013 | 1384 | 0.000 | 99 |
| **keto 1$^{twist\text{-}II}$** | -6.93 | -6.07 | 112.6 | -106.7 | 73.4 | 3.755 | 1.013 | 1390 | 0.000 | 99 |
| **keto 2** | 0.86 | 0.00 | 176.5 | 88.8 | -90.4 | 1.949 | 1.023 | 470 | 0.352 | 99 |
| **keto 2$^{twist\text{-}I}$** | -5.92 | -5.17 | -112.0 | 86.6 | -93.2 | 3.750 | 1.013 | 1386 | 0.000 | 99 |
| **keto 2$^{twist\text{-}II}$** | -6.00 | -5.02 | 112.2 | 86.3 | -93.3 | 3.759 | 1.013 | 1394 | 0.000 | 99 |
| **$H_2O$** | | | | | | | | | | |
| ***cis*-enol 1** | 6.80 | 7.31 | 179.6 | -101.4 | 77.7 | 1.010 | 1.666 | 383 | 0.800 | 98 |
| ***cis*-enol 1$^{twist\text{-}Ia}$** | 16.92 | 17.21 | -114.4 | -107.3 | 72.9 | 1.013 | 2.799 | 509 | 0.001 | 99 |
| ***cis*-enol 1$^{twist\text{-}Ib}$** | 16.39 | 16.01 | -22.1 | -86.4 | 91.3 | 0.974 | 3.816 | 396 | 0.660 | 99 |
| ***cis*-enol 1$^{twist\text{-}II}$** | 16.81 | 17.55 | 117.7 | -107.6 | 72.4 | 1.013 | 2.763 | 511 | 0.004 | 99 |
| ***cis*-enol 2** | 5.93 | 7.08 | 177.7 | 88.8 | -87.8 | 1.003 | 1.688 | 397 | 0.695 | 99 |
| ***cis*-enol 2$^{twist\text{-}Ia}$** | 17.76 | 18.22 | -112.9 | 87.6 | -92.0 | 1.013 | 2.817 | 507 | 0.000 | 98 |

| | | | | | | | | | | |
|---|---|---|---|---|---|---|---|---|---|---|
| ***cis*-enol 2$^{twist\text{-}Ib}$** | 15.72 | 15.81 | -24.2 | 88.3 | -88.3 | 0.973 | 3.808 | 398 | 0.643 | 99 |
| ***cis*-enol 2$^{twist\text{-}II}$** | 17.67 | 18.02 | 117.7 | 87.3 | -92.1 | 1.012 | 2.766 | 506 | 0.006 | 97 |
| ***trans*-enol 1** | 14.56 | 14.83 | 1.0 | 85.4 | -92.5 | 0.968 | – | 390 | 0.797 | 99 |
| ***trans*-enol 2** | 13.90 | 14.45 | -3.2 | 89.3 | -87.5 | 0.968 | – | 393 | 0.762 | 99 |
| ***trans*-enol 3** | 15.75 | 15.99 | 0.1 | 104.2 | -76.8 | 0.969 | – | 380 | 0.931 | 99 |
| **keto 1** | 0.00 | 0.00 | 178.2 | -106.9 | 73.0 | 1.955 | 1.022 | 468 | 0.347 | 99 |
| **keto 1$^{twist\text{-}I}$** | -6.57 | -4.33 | -111.8 | -107.2 | 73.0 | 3.759 | 1.013 | 1341 | 0.000 | 99 |
| **keto 1$^{twist\text{-}II}$** | -6.63 | -4.58 | 112.5 | -106.5 | 73.5 | 3.759 | 1.013 | 1346 | 0.000 | 99 |
| **keto 2** | 0.86 | 1.09 | 177.0 | 88.8 | -90.3 | 1.951 | 1.022 | 469 | 0.361 | 99 |
| **keto 2$^{twist\text{-}I}$** | -5.66 | -3.72 | -112.0 | 86.7 | -93.1 | 3.754 | 1.013 | 1343 | 0.000 | 99 |
| **keto 2$^{twist\text{-}II}$** | -5.71 | -3.52 | 112.3 | 86.3 | -93.3 | 3.763 | 1.013 | 1353 | 0.000 | 99 |

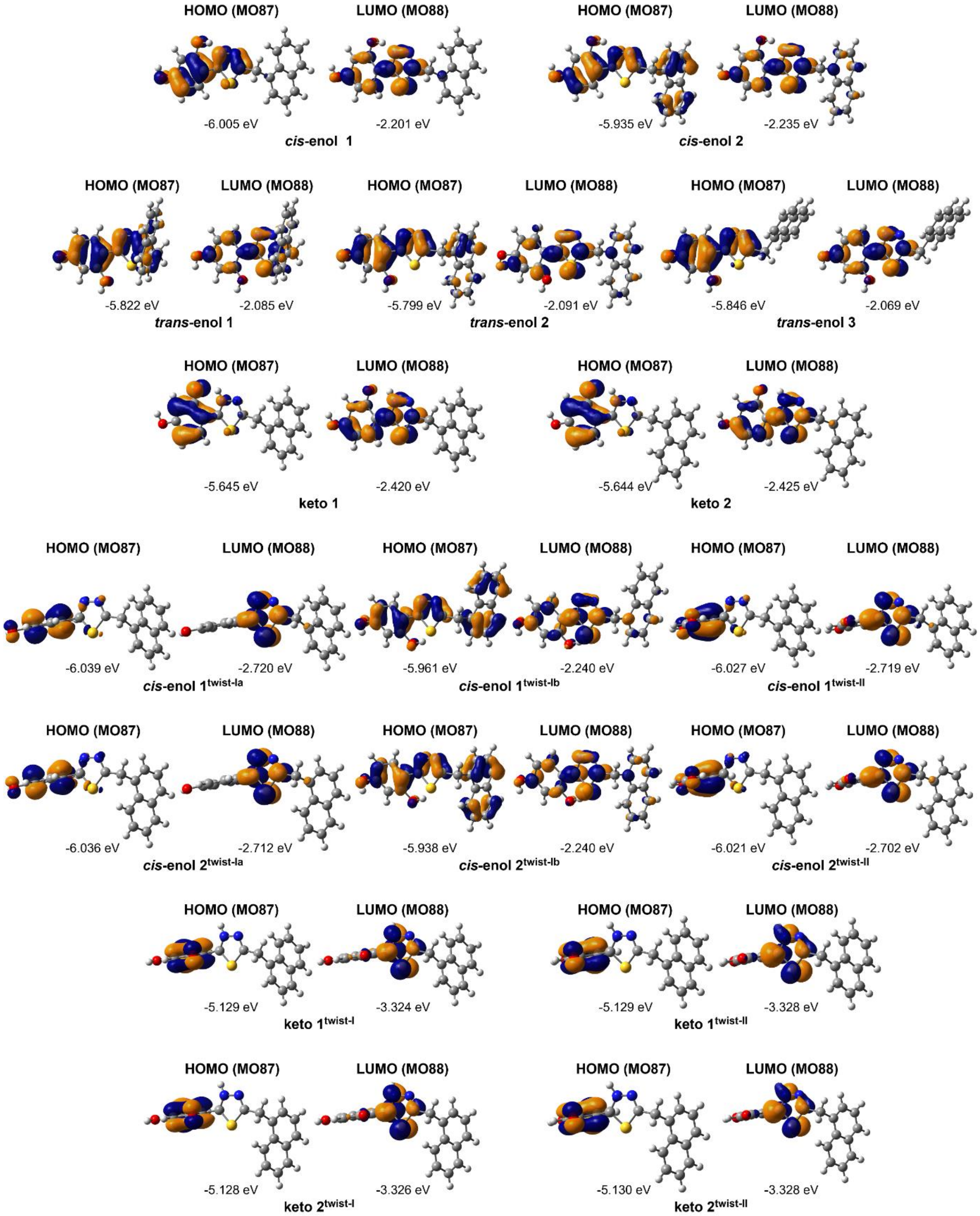


**Figure S12.** Isosurfaces (±0.04 au) of MOs of $S_0$ at $S_1$ TDDFT-optimized excited-state geometries predominantly involved in the $S_1 \rightarrow S_0$ emission transitions for different structures of enolic (*cis* and *trans*) and keto forms of NTBD, obtained based on DFT B3LYP/aug-cc-pVDZ/PCM(MeOH) calculations. Numbers listed are orbital energies. The corresponding MOs obtained from calculations with PCM($H_2O$) are almost identical, and therefore they are not displayed.

**Table S6.** Comparison of relative energy values ($\Delta E$ with respect to the particular *cis*-enol structure, in kcal/mol) and interatomic distances $d_{O\text{-}H}$ and $d_{N\text{-}H}$ as defined in Figures S8 and S11 (in Å) for structures corresponding to characteristic points on energy profiles for the proton transfer describing *cis*-enol ↔ keto tautomeric transition in the $S_0$ ground state and $S_1$ excited state of NTBD. Calculations were performed respectively at DFT ($S_0$) and TDDFT ($S_1$) B3LYP/aug-cc-pVDZ/PCM(MeOH or $H_2O$) level. Compare with Figures S13-S16.

| **Structure** | **$\Delta E$** | **$d_{O\text{-}H}$** | **$d_{N\text{-}H}$** | **$\Delta E$** | **$d_{O\text{-}H}$** | **$d_{N\text{-}H}$** |
|---|---|---|---|---|---|---|
| | | | **$S_0$** | | | |
| | | **MeOH** | | | **$H_2O$** | |
| ***cis*-enol 1** | 0.00 | 0.994 | 1.724 | 0.00 | 0.994 | 1.724 |
| **TS 1** | 7.62 | 1.375 | 1.149 | 7.57 | 1.377 | 1.149 |
| **keto 1** | 6.87 | 1.678 | 1.048 | 6.80 | 1.680 | 1.048 |
| ***cis*-enol 2** | 0.00 | 0.994 | 1.724 | 0.00 | 0.994 | 1.724 |
| **TS 2** | 7.65 | 1.376 | 1.149 | 7.61 | 1.377 | 1.149 |
| **keto 2** | 6.91 | 1.677 | 1.048 | 6.85 | 1.679 | 1.048 |
| | | | **$S_1$** | | | |
| | | **MeOH** | | | **$H_2O$** | |
| ***cis*-enol 1** | 0.00 | 1.010 | 1.665 | 0.00 | 1.010 | 1.666 |
| **TS 1** | 2.13 | 1.179 | 1.315 | 2.18 | 1.177 | 1.316 |
| **keto 1** | -6.86 | 1.953 | 1.022 | -6.80 | 1.955 | 1.022 |
| ***cis*-enol 2** | 0.00 | 1.003 | 1.688 | 0.00 | 1.003 | 1.688 |
| **TS 2** | 3.27 | 1.204 | 1.288 | 3.30 | 1.203 | 1.288 |
| **keto 2** | -5.11 | 1.949 | 1.023 | -5.07 | 1.951 | 1.022 |

**Table S7.** Comparison of relative energy values (Δ*E* with respect to the particular *cis*-enol or keto structure, in kcal/mol), dihedral angle $\angle C_1C_2C_3S$ and interatomic distances $d_{O\text{-}H}$ and $d_{N\text{-}H}$ as defined in Figure S11 (in respectively, deg and Å) for structures corresponding to selected characteristic points on energy profiles for intramolecular rotation of the resorcynyl fragment relative to the 1,3,4-thiadiazole unit (i.e. around the $C_2$-$C_3$ bond) in the *cis*-enol and keto forms of $S_1$ excited state of NTBD. Calculations were performed at TDDFT B3LYP/aug-cc-pVDZ/PCM(MeOH or $H_2O$) level. Compare with Figures S17-S24.

| **Structure** | **Δ*E*** | **$\angle C_1C_2C_3S$** | **$d_{O\text{-}H}$** | **$d_{N\text{-}H}$** | **Δ*E*** | **$\angle C_1C_2C_3S$** | **$d_{O\text{-}H}$** | **$d_{N\text{-}H}$** |
|---|---|---|---|---|---|---|---|---|
| | | | **MeOH** | | | | **$H_2O$** | |
| ***cis*-enol 1** | 0.00 | 179.6 | 1.010 | 1.665 | 0.00 | 179.6 | 1.010 | 1.666 |
| **TS Ia** | 10.28 | -139.3 | 0.995 | 2.508 | 10.40 | -139.3 | 0.994 | 2.511 |
| ***cis*-enol $1^{twist\text{-}Ia}$** | 9.98 | -114.5 | 1.014 | 2.795 | 10.12 | -114.4 | 1.013 | 2.799 |
| **TS Ib** | 10.51 | -91.3 | 1.003 | 3.097 | 10.62 | -91.2 | 1.002 | 3.100 |
| ***cis*-enol $1^{twist\text{-}Ib}$** | 9.62 | -22.5 | 0.974 | 3.812 | 9.59 | -22.1 | 0.974 | 3.816 |
| **TS II** | 10.16 | 140.1 | 0.995 | 2.506 | 10.28 | 140.0 | 0.995 | 2.509 |
| ***cis*-enol $1^{twist\text{-}II}$** | 9.86 | 117.9 | 1.014 | 2.760 | 10.02 | 117.7 | 1.013 | 2.763 |
| ***cis*-enol 2** | 0.00 | 177.7 | 1.003 | 1.688 | 0.00 | 177.7 | 1.003 | 1.688 |
| **TS Ia** | 12.03 | -141.0 | 0.993 | 2.490 | 12.10 | -140.9 | 0.993 | 2.492 |
| ***cis*-enol $2^{twist\text{-}Ia}$** | 11.72 | -113.1 | 1.014 | 2.812 | 11.83 | -112.9 | 1.013 | 2.817 |
| **TS Ib** | 11.89 | -99.4 | 1.009 | 2.987 | 11.98 | -99.3 | 1.008 | 2.989 |
| ***cis*-enol $2^{twist\text{-}Ib}$** | 9.84 | -24.3 | 0.973 | 3.807 | 9.79 | -24.2 | 0.973 | 3.808 |
| **TS II** | 11.77 | 138.1 | 0.995 | 2.534 | 11.86 | 138.0 | 0.995 | 2.536 |
| ***cis*-enol $2^{twist\text{-}II}$** | 11.63 | 117.7 | 1.013 | 2.764 | 11.75 | 117.7 | 1.012 | 2.766 |
| **keto 1** | 0.00 | 178.1 | 1.953 | 1.022 | 0.00 | 178.2 | 1.955 | 1.022 |
| **TS I** | 0.20 | -155.4 | 2.102 | 1.021 | 0.25 | -147.4 | 2.117 | 1.019 |
| **keto $1^{twist\text{-}I}$** | -6.84 | -111.9 | 3.754 | 1.013 | -6.57 | -111.8 | 3.759 | 1.013 |
| **TS II** | 0.10 | 162.8 | 2.022 | 1.021 | 0.11 | 163.1 | 2.023 | 1.021 |
| **keto $1^{twist\text{-}II}$** | -6.93 | 112.6 | 3.755 | 1.013 | -6.63 | 112.5 | 3.759 | 1.013 |
| **keto 2** | 0.00 | 176.5 | 1.949 | 1.023 | 0.00 | 177.0 | 1.951 | 1.022 |
| **TS I** | 0.18 | -162.5 | 2.026 | 1.021 | 0.20 | -162.4 | 2.032 | 1.021 |
| **keto $2^{twist\text{-}I}$** | -6.79 | -112.0 | 3.750 | 1.013 | -6.52 | -112.0 | 3.754 | 1.013 |
| **TS II** | 0.20 | 147.7 | 2.208 | 1.019 | 0.25 | 150.1 | 2.195 | 1.019 |
| **keto $2^{twist\text{-}II}$** | -6.87 | 112.2 | 3.759 | 1.013 | -6.57 | 112.3 | 3.763 | 1.013 |

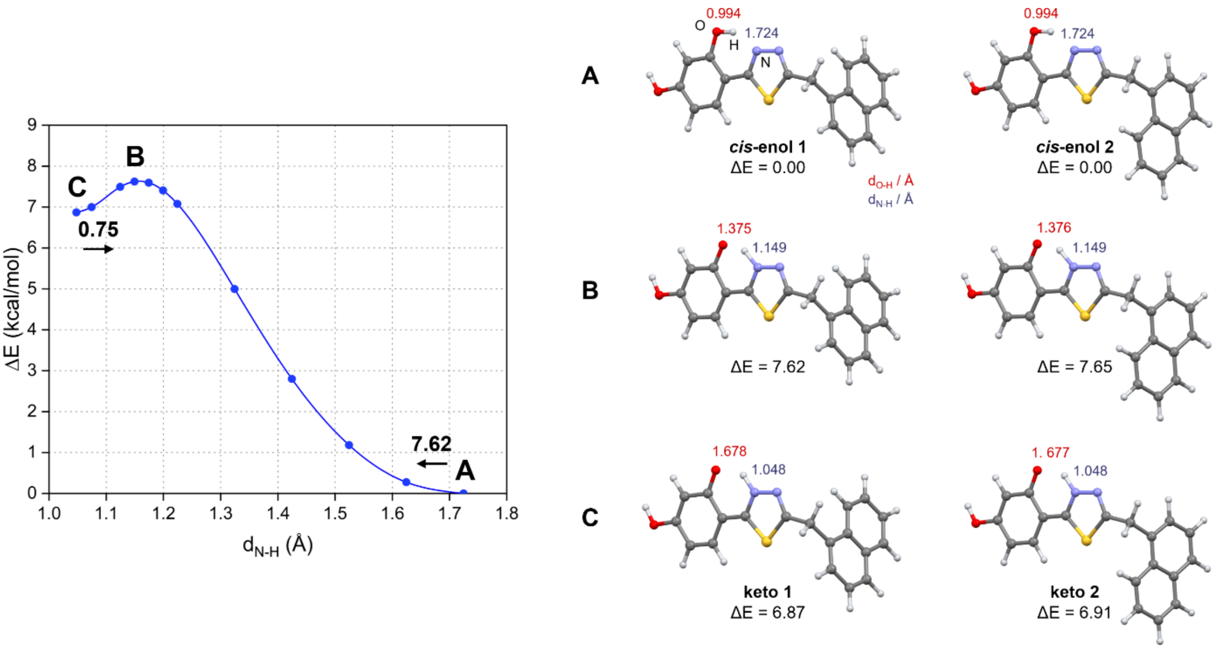


**Figure S13.** Left panel: Energy profile for the proton transfer describing *cis*-enol 1 ↔ keto 1 tautomeric transition in the ground state of NTBD modelled in MeOH. Numbers listed along arrows displayed in the chart are estimated energy barriers (in kcal/mol) of the transition between two adjacent minima in the direction indicated by the arrow. Right panel: Molecular structures corresponding to characteristic points (A: *cis*-enol, C: keto, B: approximate transition state) on the tautomerization profile computed for both *cis*-enol 1 ↔ keto 1 and *cis*-enol 2 ↔ keto 2 pathways. Values listed are relative energy Δ*E* with respect to the particular *cis*-enol structure (in kcal/mol) and interatomic distances $d_{O\text{-}H}$ and $d_{N\text{-}H}$ as defined on the *cis*-enol 1 geometry. Calculations were performed at DFT B3LYP/aug-cc-pVDZ/PCM(MeOH) level.

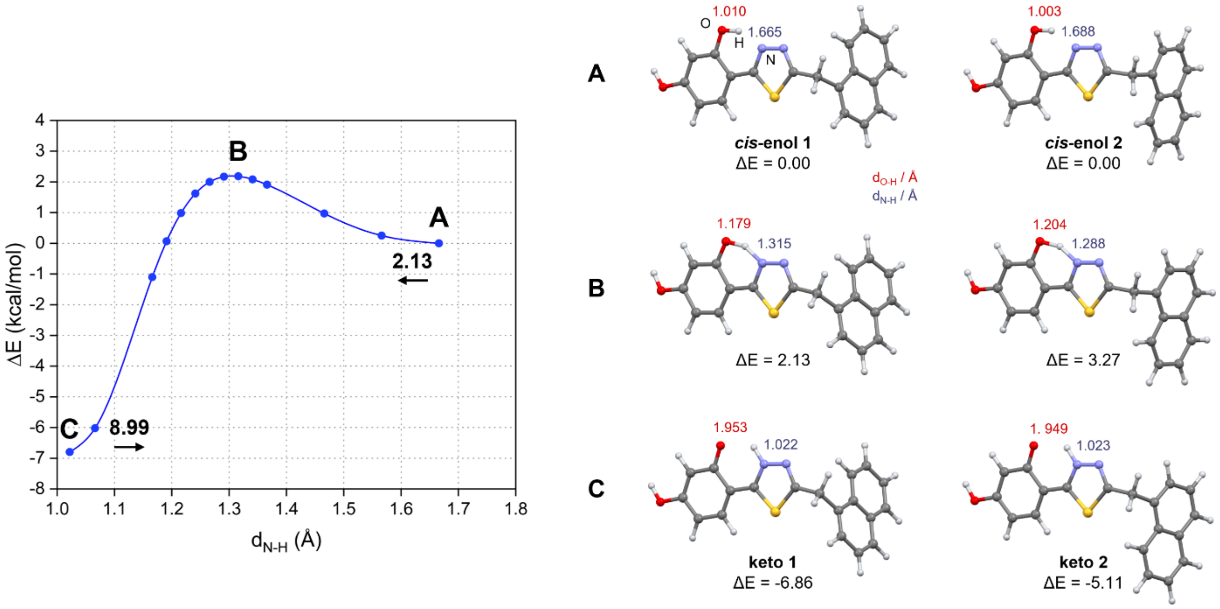


**Figure S14.** Left panel: Energy profile for the proton transfer describing *cis*-enol 1 ↔ keto 1 tautomeric transition in the $S_1$ excited state of NTBD modelled in MeOH. Numbers listed along arrows displayed in the chart are estimated energy barriers (in kcal/mol) of the transition between two adjacent minima in the direction indicated by the arrow. Right panel: Molecular structures corresponding to characteristic points (A: *cis*-enol, C: keto, B: approximate transition state) on the tautomerization profile computed for both *cis*-enol 1 ↔ keto 1 and *cis*-enol 2 ↔ keto 2 ESIPT pathways. Values listed are relative energy Δ*E* with respect to the particular *cis*-enol structure (in kcal/mol) and interatomic distances $d_{O\text{-}H}$ and $d_{N\text{-}H}$ as defined on the *cis*-enol 1 geometry. Calculations were performed at TDDFT B3LYP/aug-cc-pVDZ/PCM(MeOH) level.

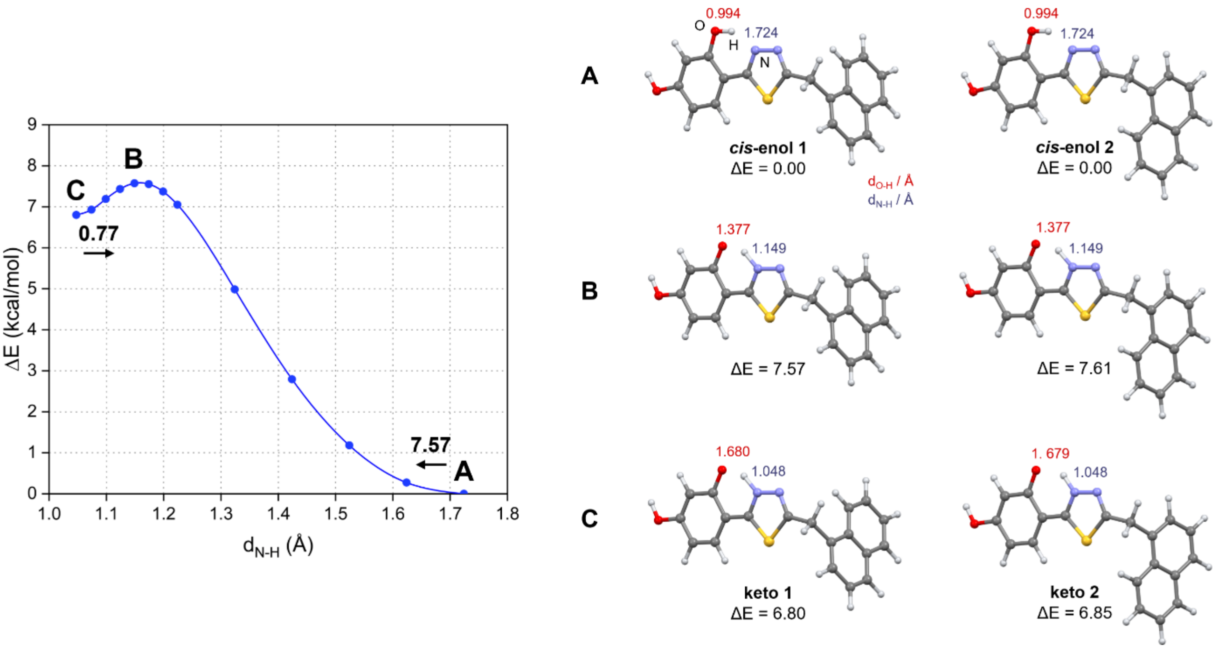


**Figure S15.** Left panel: Energy profile for the proton transfer describing *cis*-enol 1 ↔ keto 1 tautomeric transition in the ground state of NTBD modelled in $H_2O$. Numbers listed along arrows displayed in the chart are estimated energy barriers (in kcal/mol) of the transition between two adjacent minima in the direction indicated by the arrow. Right panel: Molecular structures corresponding to characteristic points (A: *cis*-enol, C: keto, B: approximate transition state) on the tautomerization profile computed for both *cis*-enol 1 ↔ keto 1 and *cis*-enol 2 ↔ keto 2 pathways. Values listed are relative energy Δ*E* with respect to the particular *cis*-enol structure (in kcal/mol) and interatomic distances $d_{O\text{-}H}$ and $d_{N\text{-}H}$ as defined on the *cis*-enol 1 geometry. Calculations were performed at DFT B3LYP/aug-cc-pVDZ/PCM($H_2O$) level.

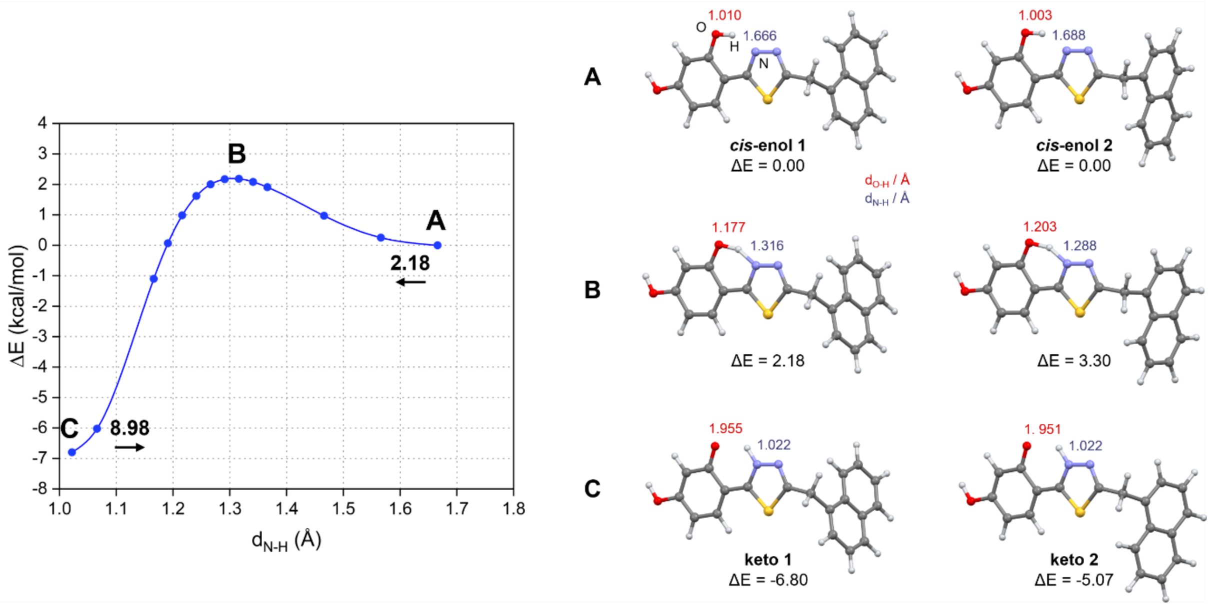


**Figure S16.** Left panel: Energy profile for the proton transfer describing *cis*-enol 1 ↔ keto 1 tautomeric transition in the $S_1$ excited state of NTBD modelled in $H_2O$. Numbers listed along arrows displayed in the chart are estimated energy barriers (in kcal/mol) of the transition between two adjacent minima in the direction indicated by the arrow. Right panel: Molecular structures corresponding to characteristic points (A: *cis*-enol, C: keto, B: approximate transition state) on the tautomerization profile computed for both *cis*-enol 1 ↔ keto 1 and *cis*-enol 2 ↔ keto 2 ESIPT pathways. Values listed are relative energy $\Delta E$ with respect to the particular *cis*-enol structure (in kcal/mol) and interatomic distances $d_{O\text{-}H}$ and $d_{N\text{-}H}$ as defined on the *cis*-enol 1 geometry. Calculations were performed at TDDFT B3LYP/aug-cc-pVDZ/PCM($H_2O$) level.

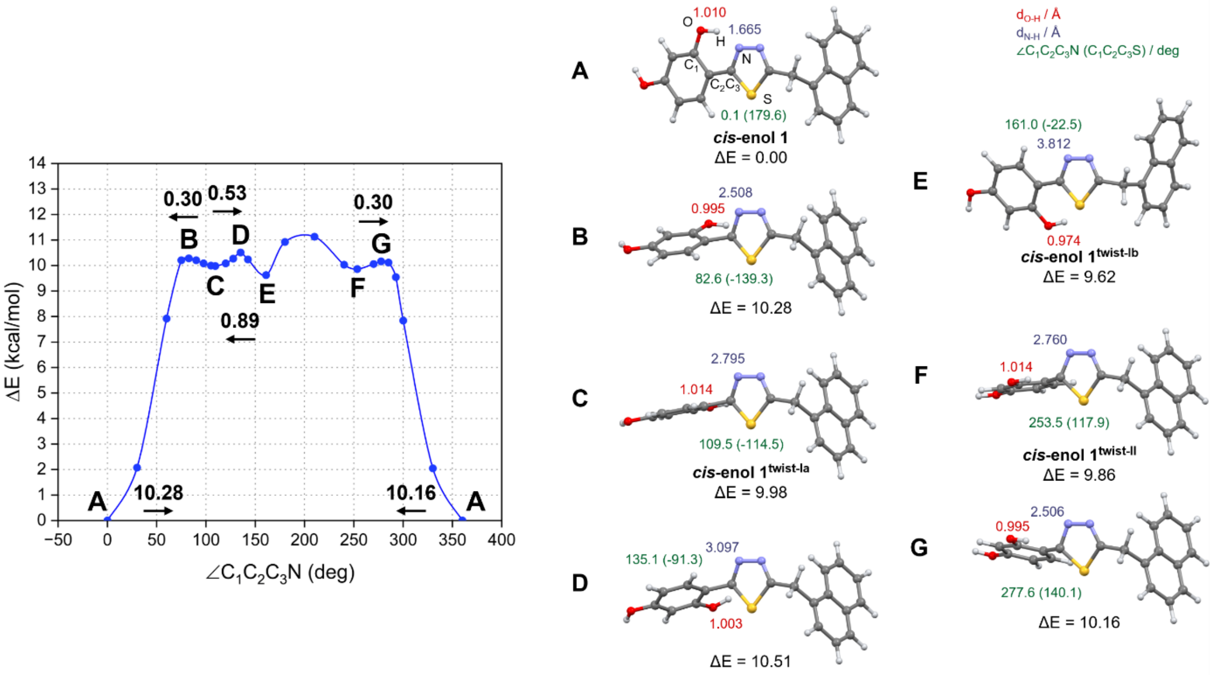


**Figure S17.** Energy profile for the rotation of the resorcynyl fragment relative to the 1,3,4-thiadiazole unit (i.e. around the $C_2$-$C_3$ bond corresponding to the change of the $C_1C_2C_3N$ dihedral angle from 0 to 360 deg; see *cis*-enol 1 structure for atoms labelling) in the $S_1$ excited-state *cis*-enol 1 structure of NTBD (left panel) along with molecular structures corresponding to its characteristic points (A: planar *cis*-enol, C, E and F: twisted *cis*-enol, B, D and G: approximate transition state, right panel). Numbers listed along arrows displayed in the chart are estimated energy barriers (in kcal/mol) of the transition between two adjacent minima in the direction indicated by the arrow. Values listed in right panel are relative energy Δ*E* with respect to the planar *cis*-enol structure (in kcal/mol), interatomic distances $d_{O\text{-}H}$ and $d_{N\text{-}H}$, and dihedral angles $\angle C_1C_2C_3N$ and $\angle C_1C_2C_3S$ as defined on the *cis*-enol 1 geometry. Based on TDDFT B3LYP/aug-cc-pVDZ/PCM(MeOH) calculations.

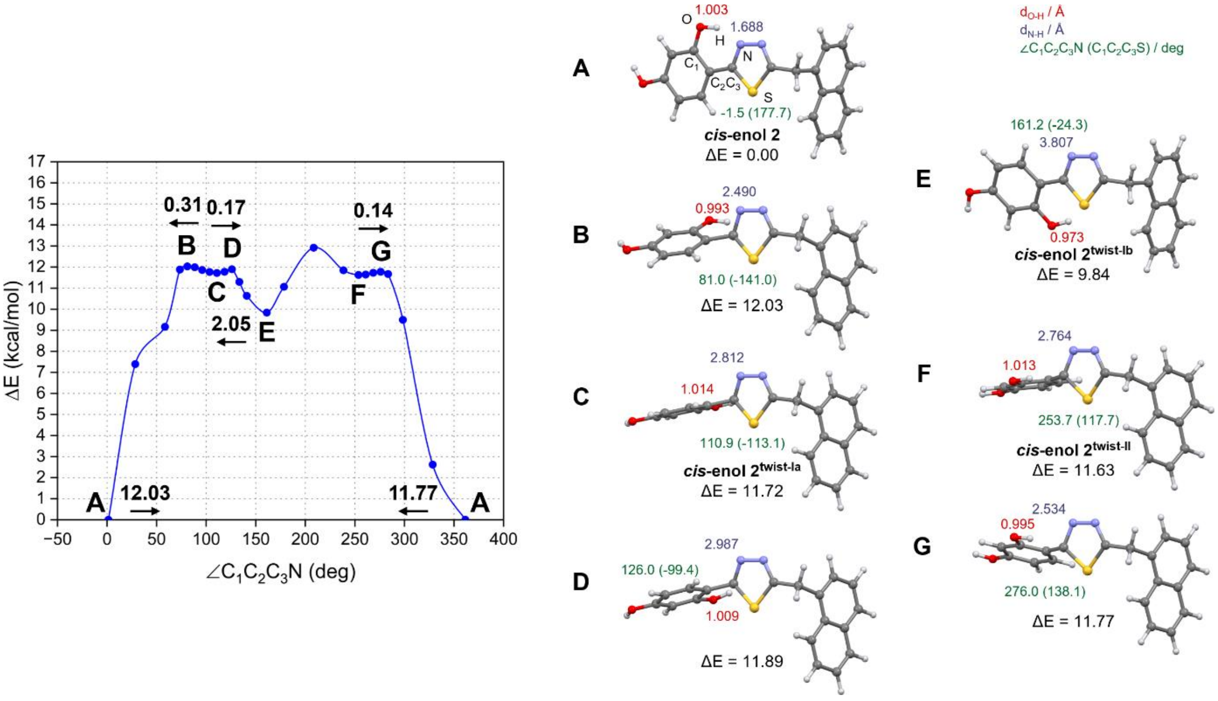


**Figure S18.** Energy profile for the rotation of the resorcynyl fragment relative to the 1,3,4-thiadiazole unit (i.e. around the $C_2$-$C_3$ bond corresponding to the change of the $C_1C_2C_3N$ dihedral angle from 0 to 360 deg; see *cis*-enol 2 structure for atoms labelling) in the $S_1$ excited-state *cis*-enol 2 structure of NTBD (left panel) along with molecular structures corresponding to its characteristic points (A: planar *cis*-enol, C, E and F: twisted *cis*-enol, B, D and G: approximate transition state, right panel). Numbers listed along arrows displayed in the chart are estimated energy barriers (in kcal/mol) of the transition between two adjacent minima in the direction indicated by the arrow. Values listed in right panel are relative energy $\Delta E$ with respect to the planar *cis*-enol structure (in kcal/mol), interatomic distances $d_{O\text{-}H}$ and $d_{N\text{-}H}$, and dihedral angles $\angle C_1C_2C_3N$ and $\angle C_1C_2C_3S$ as defined on the *cis*-enol 2 geometry. Based on TDDFT B3LYP/aug-cc-pVDZ/PCM(MeOH) calculations.

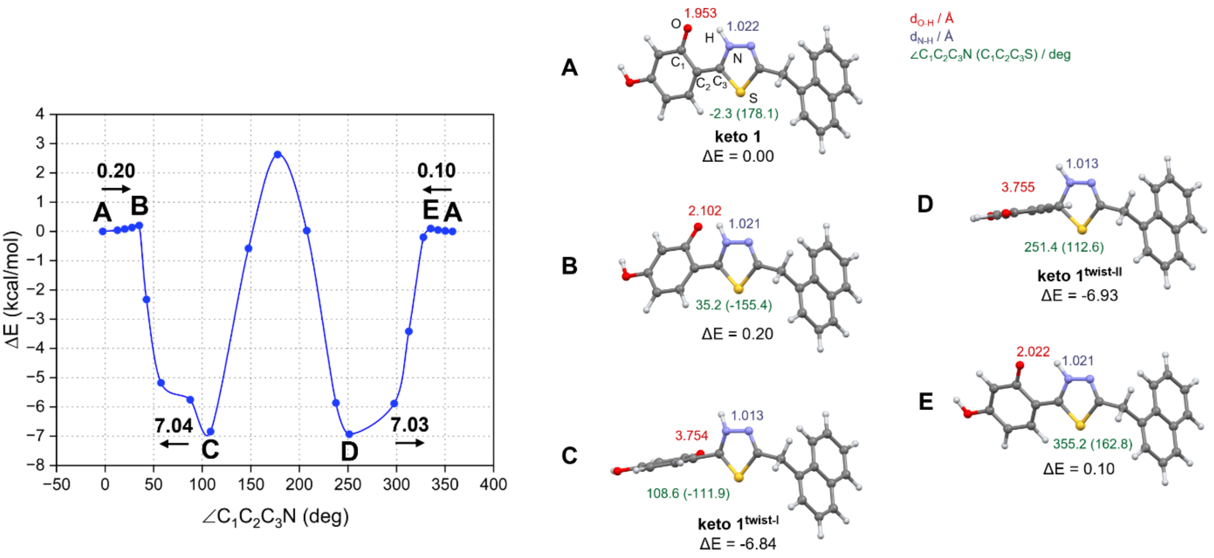


**Figure S19.** Energy profile for the rotation of the resorcynyl fragment relative to the 1,3,4-thiadiazole unit (i.e. around the $C_2$-$C_3$ bond corresponding to the change of the $C_1C_2C_3N$ dihedral angle from 0 to 360 deg; see keto 1 structure for atoms labelling) in the $S_1$ excited-state keto 1 structure of NTBD (left panel) along with molecular structures corresponding to its characteristic points (A: planar keto, C and D: twisted keto, B and E: approximate transition state, right panel). Numbers listed along arrows displayed in the chart are estimated energy barriers (in kcal/mol) of the transition between two adjacent minima in the direction indicated by the arrow. Values listed in right panel are relative energy Δ*E* with respect to the planar keto structure (in kcal/mol), interatomic distances $d_{O\text{-}H}$ and $d_{N\text{-}H}$, and dihedral angles $\angle C_1C_2C_3N$ and $\angle C_1C_2C_3S$ as defined on the keto 1 geometry. Based on TDDFT B3LYP/aug-cc-pVDZ/PCM(MeOH) calculations.

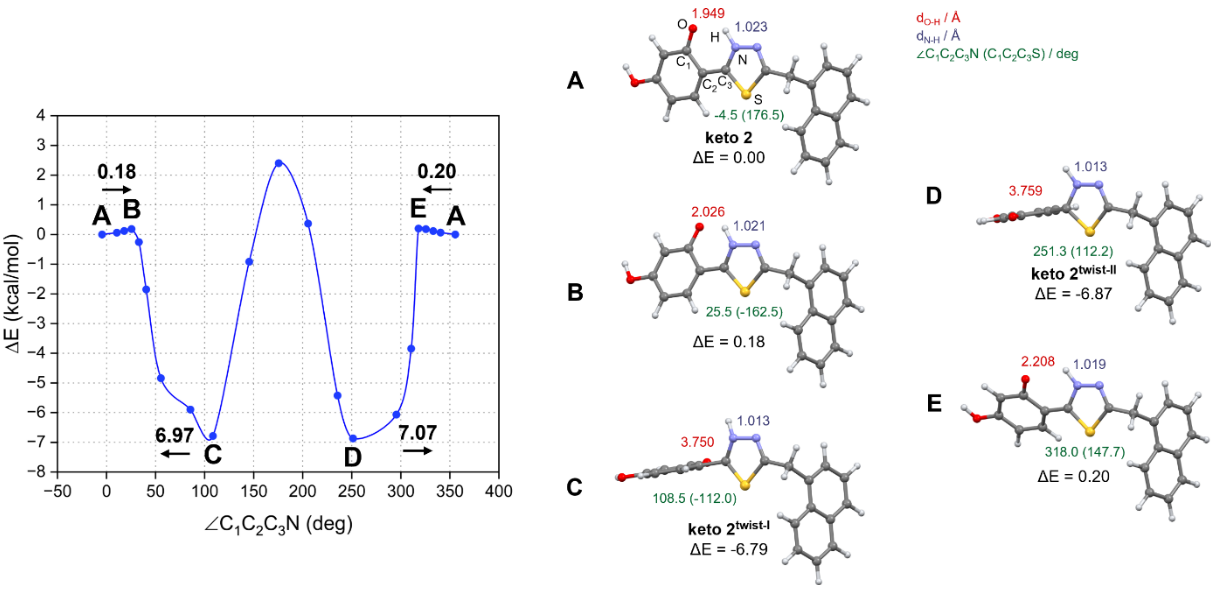


**Figure S20.** Energy profile for the rotation of the resorcynyl fragment relative to the 1,3,4-thiadiazole unit (i.e. around the $C_2$-$C_3$ bond corresponding to the change of the $C_1C_2C_3N$ dihedral angle from 0 to 360 deg; see keto 2 structure for atoms labelling) in the $S_1$ excited-state keto 2 structure of NTBD (left panel) along with molecular structures corresponding to its characteristic points (A: planar keto, C and D: twisted keto, B and E: approximate transition state, right panel). Numbers listed along arrows displayed in the chart are estimated energy barriers (in kcal/ mol) of the transition between two adjacent minima in the direction indicated by the arrow. Values listed in right panel are relative energy $\Delta E$ with respect to the planar keto structure (in kcal/mol), interatomic distances $d_{O\text{-}H}$ and $d_{N\text{-}H}$, and dihedral angles $\angle C_1C_2C_3N$ and $\angle C_1C_2C_3S$ as defined on the keto 2 geometry. Based on TDDFT B3LYP/aug-cc-pVDZ/PCM(MeOH) calculations.

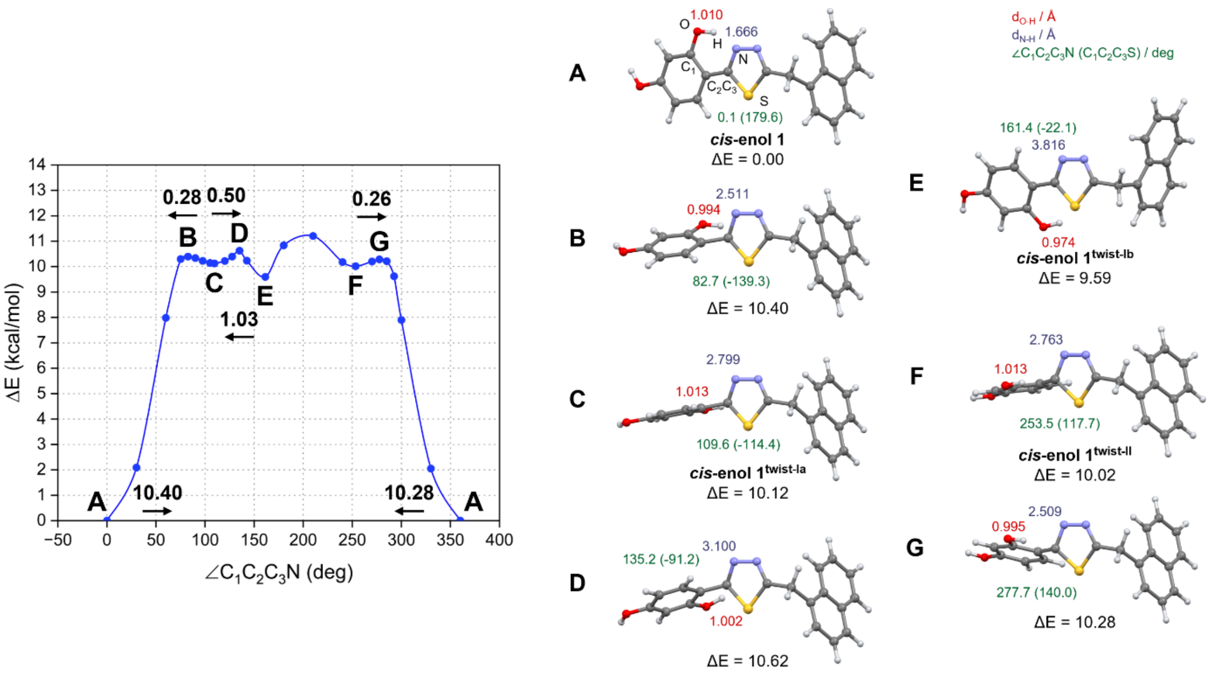


**Figure S21.** Energy profile for the rotation of the resorcynyl fragment relative to the 1,3,4-thiadiazole unit (i.e. around the $C_2$-$C_3$ bond corresponding to the change of the $C_1C_2C_3N$ dihedral angle from 0 to 360 deg; see *cis*-enol 1 structure for atoms labelling) in the $S_1$ excited-state *cis*-enol 1 structure of NTBD (left panel) along with molecular structures corresponding to its characteristic points (A: planar *cis*-enol, C, E and F: twisted *cis*-enol, B, D and G: approximate transition state, right panel). Numbers listed along arrows displayed in the chart are estimated energy barriers (in kcal/mol) of the transition between two adjacent minima in the direction indicated by the arrow. Values listed in right panel are relative energy Δ*E* with respect to the planar *cis*-enol structure (in kcal/mol), interatomic distances $d_{O\text{-}H}$ and $d_{N\text{-}H}$, and dihedral angles $\angle C_1C_2C_3N$ and $\angle C_1C_2C_3S$ as defined on the *cis*-enol 1 geometry. Based on TDDFT B3LYP/aug-cc-pVDZ/PCM($H_2O$) calculations.

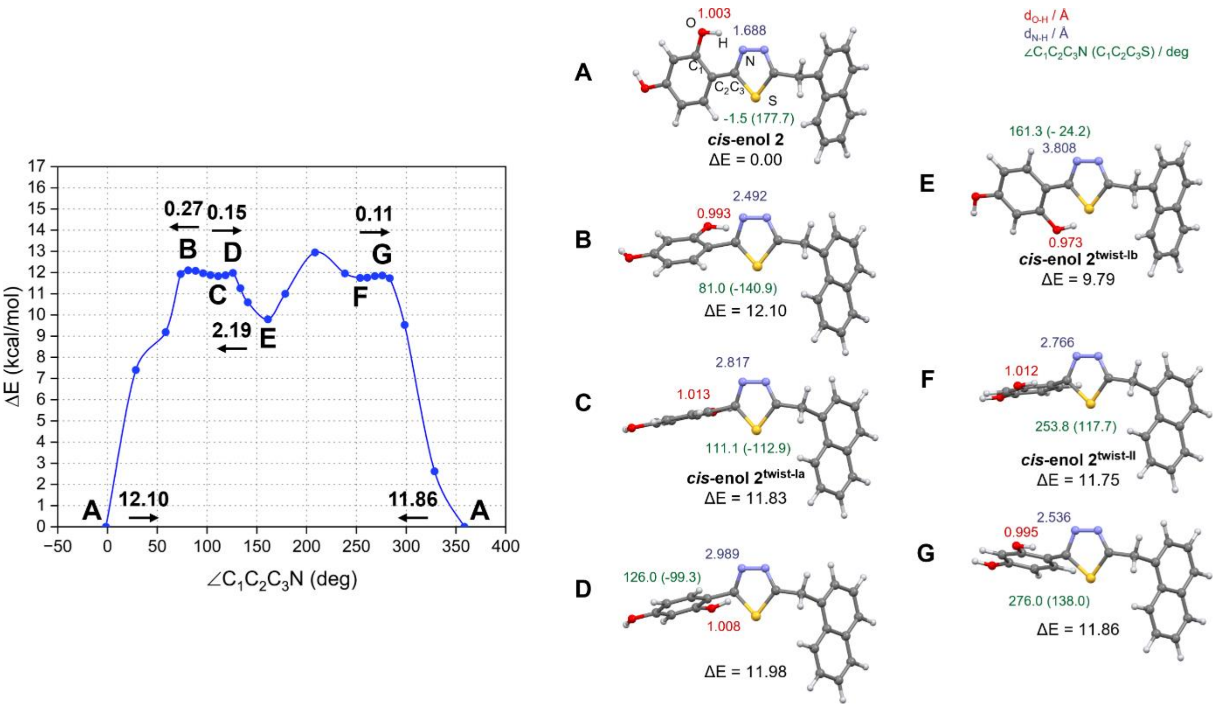


**Figure S22.** Energy profile for the rotation of the resorcynyl fragment relative to the 1,3,4-thiadiazole unit (i.e. around the $C_2$-$C_3$ bond corresponding to the change of the $C_1C_2C_3N$ dihedral angle from 0 to 360 deg; see *cis*-enol 2 structure for atoms labelling) in the $S_1$ excited-state *cis*-enol 2 structure of NTBD (left panel) along with molecular structures corresponding to its characteristic points (A: planar *cis*-enol, C, E and F: twisted *cis*-enol, B, D and G: approximate transition state, right panel). Numbers listed along arrows displayed in the chart are estimated energy barriers (in kcal/mol) of the transition between two adjacent minima in the direction indicated by the arrow. Values listed in right panel are relative energy $\Delta E$ with respect to the planar *cis*-enol structure (in kcal/mol), interatomic distances $d_{O\text{-}H}$ and $d_{N\text{-}H}$, and dihedral angles $\angle C_1C_2C_3N$ and $\angle C_1C_2C_3S$ as defined on the *cis*-enol 2 geometry. Based on TDDFT B3LYP/aug-cc-pVDZ/PCM($H_2O$) calculations.

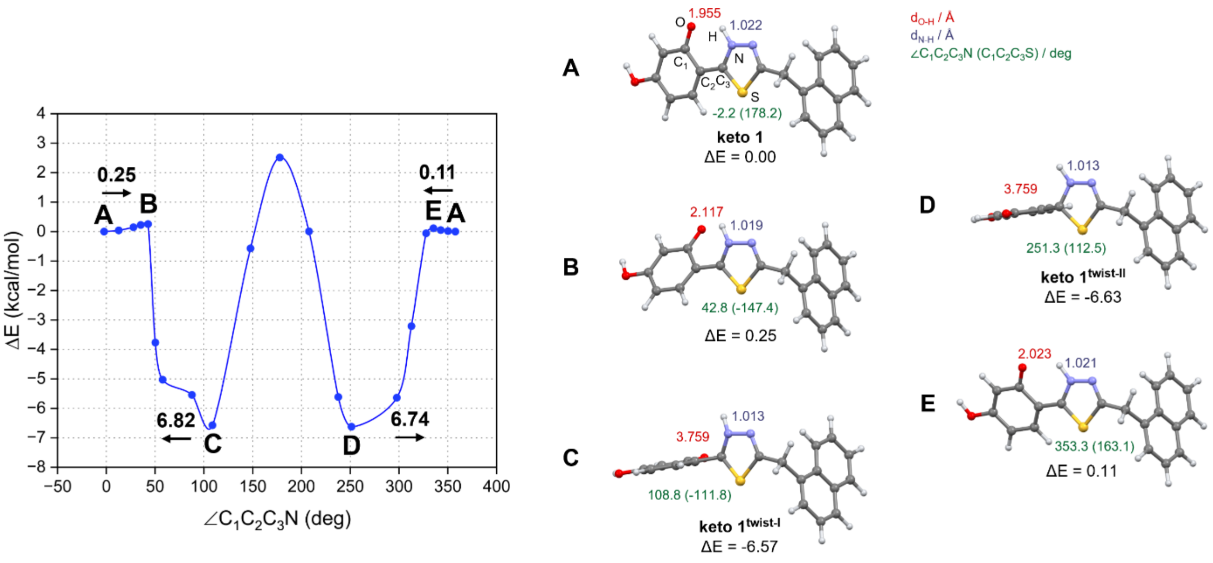


**Figure S23.** Energy profile for the rotation of the resorcynyl fragment relative to the 1,3,4-thiadiazole unit (i.e. around the $C_2$-$C_3$ bond corresponding to the change of the $C_1C_2C_3N$ dihedral angle from 0 to 360 deg; see keto 1 structure for atoms labelling) in the $S_1$ excited-state keto 1 structure of NTBD (left panel) along with molecular structures corresponding to its characteristic points (A: planar keto, C and D: twisted keto, B and E: approximate transition state, right panel). Numbers listed along arrows displayed in the chart are estimated energy barriers (in kcal/mol) of the transition between two adjacent minima in the direction indicated by the arrow. Values listed in right panel are relative energy $\Delta E$ with respect to the planar keto structure (in kcal/mol), interatomic distances $d_{O\text{-}H}$ and $d_{N\text{-}H}$, and dihedral angles $\angle C_1C_2C_3N$ and $\angle C_1C_2C_3S$ as defined on the keto 1 geometry. Based on TDDFT B3LYP/aug-cc-pVDZ/PCM($H_2O$) calculations.

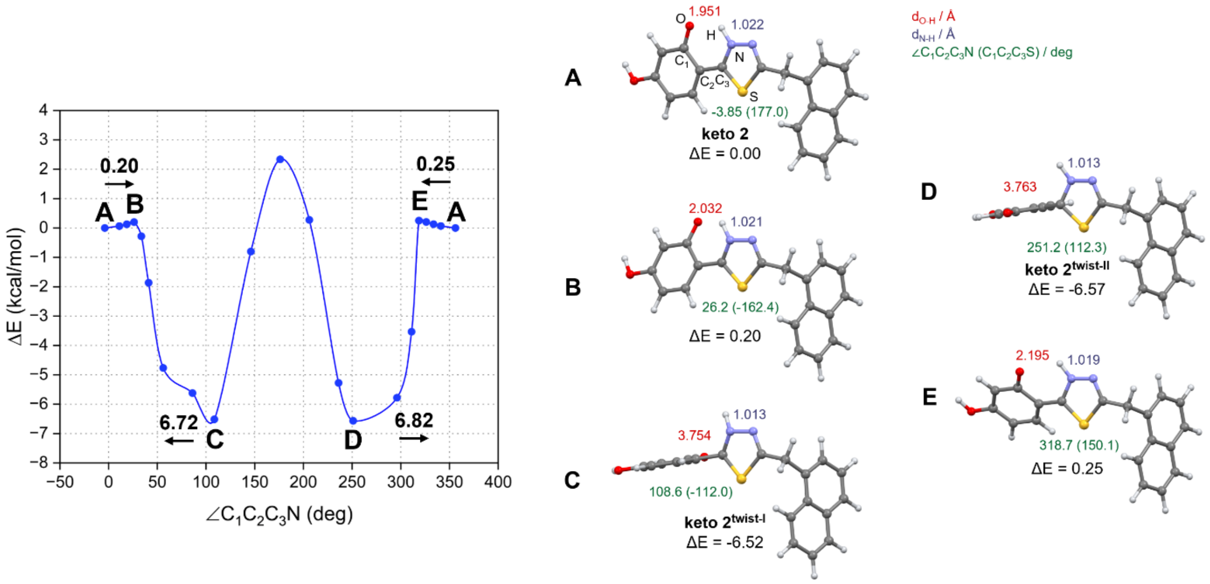


**Figure S24.** Energy profile for the rotation of the resorcynyl fragment relative to the 1,3,4-thiadiazole unit (i.e. around the $C_2$-$C_3$ bond corresponding to the change of the $C_1C_2C_3N$ dihedral angle from 0 to 360 deg; see keto 2 structure for atoms labelling) in the $S_1$ excited-state keto 2 structure of NTBD (left panel) along with molecular structures corresponding to its characteristic points (A: planar keto, C and D: twisted keto, B and E: approximate transition state, right panel). Numbers listed along arrows displayed in the chart are estimated energy barriers (in kcal/mol) of the transition between two adjacent minima in the direction indicated by the arrow. Values listed in right panel are relative energy $\Delta E$ with respect to the planar keto structure (in kcal/mol), interatomic distances $d_{O\text{-}H}$ and $d_{N\text{-}H}$, and dihedral angles $\angle C_1C_2C_3N$ and $\angle C_1C_2C_3S$ as defined on the keto 2 geometry. Based on TDDFT B3LYP/aug-cc-pVDZ/PCM($H_2O$) calculations.

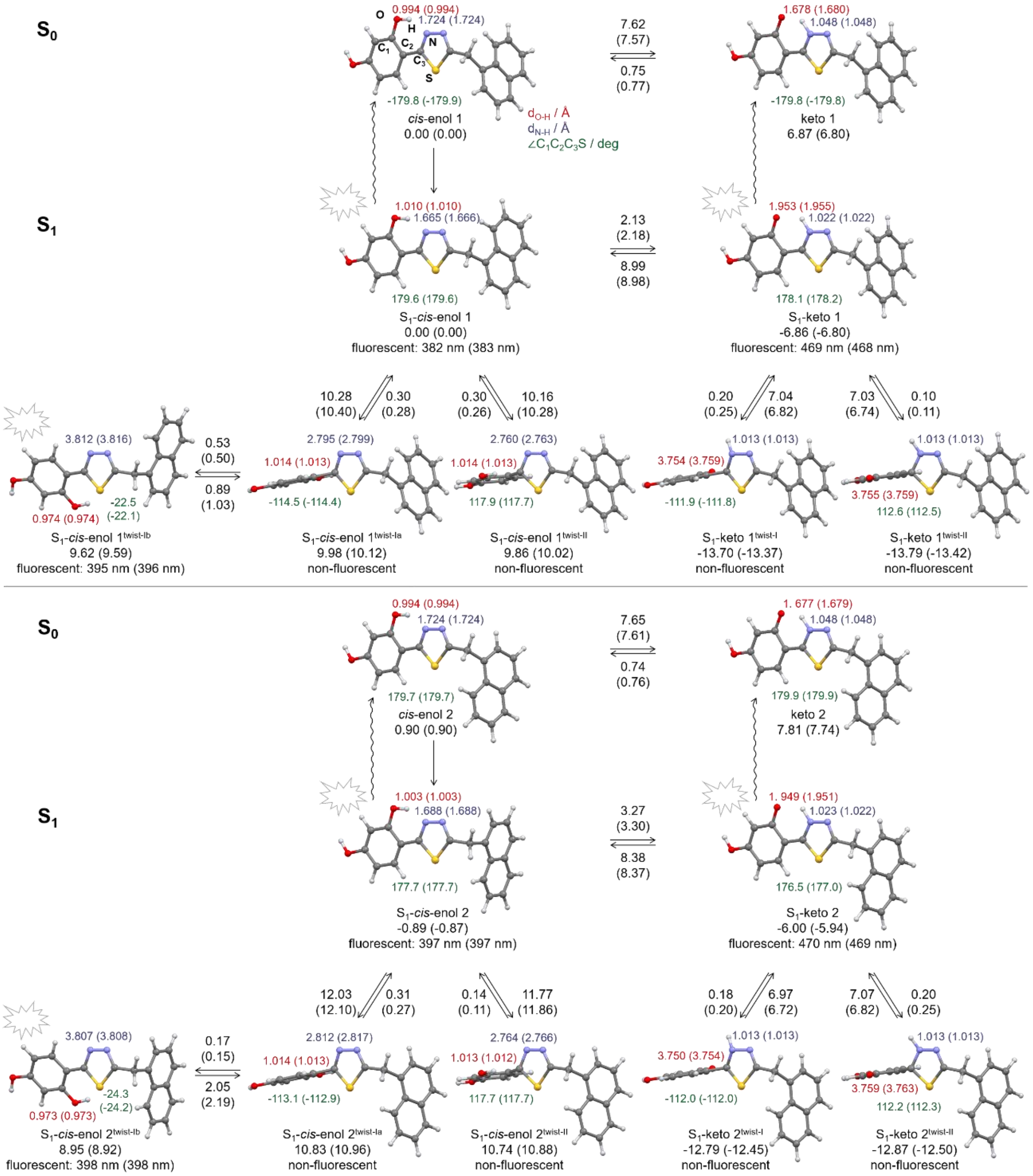


**Figure S25.** Low-energy optimized structures of *cis*-enol and keto NTBD tautomers (1 – top, 2 – bottom) in the $S_0$ ground state (top of each panel) and in the $S_1$ excited state (bottom of each panel). Numbers displayed along arrows are estimated energy barriers (in kcal/mol) of the forward and reverse transitions between two adjacent minima in the direction indicated by the arrow with respect to energy profiles connecting given structures. Other numbers listed are relative energy values with respect to the *cis*-enol 1 structure, in kcal/mol, interatomic O-H and N-H distances along with C-C-C-S dihedral angle as defined on the *cis*-enol 1 geometry, and for fluorescent structures TDDFT $S_1$-$S_0$ energy differences representing emission wavelengths. Based on B3LYP/aug-cc-pVDZ/PCM(MeOH) calculations. Values provided in parentheses correspond to the results obtained with PCM($H_2O$).

**Cartesian coordinates for optimized structures**

Selected DFT-optimized (B3LYP+D3/6-311++G(d,p) with continuum solvent model for MeOH) geometries of low-energy structures of NTBD in its enolic (*cis* and *trans*) and keto forms along with the corresponding absolute energies.

The atomic symbol followed by three Cartesian coordinates, in Å.

*cis*-enol 1

Total energy = -1390.993548 au

| | | | |
|---|---|---|---|
| C | 0.599524 | -0.198220 | -0.262280 |
| C | 0.873851 | -1.557292 | -0.025757 |
| C | 2.163742 | -2.043976 | 0.029659 |
| C | 3.233460 | -1.155801 | -0.155570 |
| C | 3.002574 | 0.193956 | -0.394885 |
| C | 1.696108 | 0.682999 | -0.450223 |
| H | 0.049597 | -2.246063 | 0.117360 |
| H | 2.362891 | -3.091369 | 0.212830 |
| O | 4.488407 | -1.677490 | -0.090317 |
| H | 3.824161 | 0.885702 | -0.540740 |
| O | 1.534591 | 2.000049 | -0.684029 |
| C | -0.757300 | 0.310059 | -0.310987 |
| S | -2.199275 | -0.651666 | -0.052382 |
| C | -3.115424 | 0.822013 | -0.308481 |
| N | -2.374602 | 1.862304 | -0.538190 |
| N | -1.039533 | 1.573368 | -0.545227 |
| C | -4.614729 | 0.842085 | -0.259726 |
| H | 0.562612 | 2.197777 | -0.697428 |
| H | 5.147201 | -0.985044 | -0.224487 |
| C | -5.187105 | -0.046354 | 0.823154 |
| H | -4.920191 | 1.883534 | -0.133398 |
| H | -4.997844 | 0.513259 | -1.229384 |
| C | -5.882934 | -1.183326 | 0.476647 |
| C | -6.405583 | -2.055849 | 1.458770 |
| C | -6.222811 | -1.781678 | 2.790519 |
| C | -5.512996 | -0.621305 | 3.197163 |
| C | -4.986400 | 0.265817 | 2.204269 |
| H | -6.030234 | -1.417618 | -0.571773 |
| H | -6.947937 | -2.941674 | 1.150352 |
| H | -6.616772 | -2.446370 | 3.551307 |
| C | -5.312583 | -0.321988 | 4.569676 |
| C | -4.278732 | 1.418844 | 2.636837 |
| C | -4.101104 | 1.679790 | 3.975898 |
| C | -4.621084 | 0.801912 | 4.953758 |
| H | -5.716364 | -1.000476 | 5.313128 |
| H | -4.473094 | 1.019204 | 6.005006 |
| H | -3.868834 | 2.103757 | 1.906518 |
| H | -3.558010 | 2.565210 | 4.284928 |

*cis*-enol 2

Total energy = -1390.991787 au

| | | | |
|---|---|---|---|
| C | 0.136173 | 0.189650 | -0.225630 |
| C | 0.674014 | -0.467426 | 0.911654 |
| C | 1.532229 | 0.218009 | 1.773301 |
| C | 1.863427 | 1.544103 | 1.520442 |
| C | 1.342163 | 2.212154 | 0.402580 |
| C | 0.493870 | 1.531879 | -0.446843 |
| O | 0.390618 | -1.750852 | 1.207799 |
| H | 1.930588 | -0.304031 | 2.635482 |
| O | 2.696646 | 2.251281 | 2.330956 |
| H | 1.610657 | 3.244426 | 0.222042 |
| H | 0.093402 | 2.050820 | -1.309760 |
| C | -0.752142 | -0.523060 | -1.122573 |
| S | -1.479169 | 0.156692 | -2.565158 |
| C | -2.242999 | -1.406060 | -2.807917 |
| N | -1.941015 | -2.275770 | -1.891595 |
| N | -1.101525 | -1.776903 | -0.936799 |
| C | -3.135996 | -1.712808 | -3.974429 |
| C | -2.472658 | -1.430514 | -5.308554 |
| H | -3.381967 | -2.772545 | -3.900926 |
| H | -4.073012 | -1.161275 | -3.870930 |
| C | -2.546958 | -0.141284 | -5.921250 |
| C | -1.844531 | 0.078845 | -7.151824 |
| C | -1.108242 | -0.983457 | -7.736177 |
| C | -1.063915 | -2.215273 | -7.132311 |
| C | -1.746786 | -2.432203 | -5.915341 |
| C | -3.280600 | 0.942924 | -5.367250 |
| C | -1.899721 | 1.358415 | -7.763436 |
| H | -0.582710 | -0.804241 | -8.667521 |
| H | -0.504266 | -3.026836 | -7.581924 |
| H | -1.694434 | -3.408155 | -5.445580 |
| C | -3.311611 | 2.171624 | -5.984069 |
| C | -2.612786 | 2.385227 | -7.193627 |
| H | -1.364267 | 1.510883 | -8.694090 |
| H | -2.645320 | 3.358141 | -7.669527 |
| H | -3.878462 | 2.982044 | -5.541216 |
| H | -3.831776 | 0.808511 | -4.445809 |
| H | -0.223132 | -2.103366 | 0.512815 |
| H | 3.000037 | 1.700433 | 3.063180 |

keto 1

Total energy = -1390.983264 au

| | | | |
|---|---|---|---|
| C | -4.620390 | 0.797153 | 4.956760 |
| C | -5.297386 | -0.332307 | 4.563253 |
| C | -5.494790 | -0.621946 | 3.188393 |
| C | -4.981100 | 0.281097 | 2.203206 |
| C | -4.289137 | 1.439797 | 2.645314 |
| C | -4.113398 | 1.690742 | 3.986549 |
| C | -5.177864 | -0.023692 | 0.820074 |
| C | -5.856753 | -1.167615 | 0.463043 |
| C | -6.367626 | -2.054886 | 1.437978 |
| C | -6.188487 | -1.788455 | 2.771738 |
| C | -4.611921 | 0.877381 | -0.256013 |
| C | -3.114541 | 0.849134 | -0.304935 |

| | | | |
|---|---|---|---|
| S | -2.219465 | -0.652927 | -0.030133 |
| C | -0.742399 | 0.238614 | -0.291471 |
| N | -1.057947 | 1.517659 | -0.537948 |
| N | -2.368100 | 1.873616 | -0.542841 |
| C | 0.585437 | -0.234894 | -0.250349 |
| C | 0.874397 | -1.598415 | 0.007303 |
| C | 2.160732 | -2.063037 | 0.055744 |
| C | 3.224285 | -1.147730 | -0.157995 |
| C | 2.993829 | 0.185748 | -0.413946 |
| C | 1.668956 | 0.716830 | -0.475172 |
| O | 4.478226 | -1.675799 | -0.093345 |
| O | 1.450093 | 1.951644 | -0.711683 |
| H | 0.056650 | -2.292336 | 0.169102 |
| H | 2.385271 | -3.102813 | 0.251712 |
| H | 3.819485 | 0.870432 | -0.575704 |
| H | 5.135853 | -0.985785 | -0.246829 |
| H | -4.915487 | 1.918012 | -0.120825 |
| H | -4.993373 | 0.556299 | -1.228814 |
| H | -5.999712 | -1.395689 | -0.587293 |
| H | -6.897381 | -2.945688 | 1.122436 |
| H | -6.572848 | -2.464893 | 3.527016 |
| H | -5.691422 | -1.022699 | 5.300865 |
| H | -4.474168 | 1.006975 | 6.009718 |
| H | -3.891007 | 2.138898 | 1.921908 |
| H | -3.582440 | 2.580813 | 4.303022 |
| H | -0.263868 | 2.168123 | -0.693382 |

keto 2

Total energy = -1390.981473 au

| | | | |
|---|---|---|---|
| C | 0.133333 | 0.209674 | -0.251371 |
| C | 0.628677 | -0.518981 | 0.912371 |
| C | 1.502400 | 0.204330 | 1.780841 |
| C | 1.851268 | 1.512020 | 1.526034 |
| C | 1.361318 | 2.209070 | 0.390728 |
| C | 0.520909 | 1.555617 | -0.469257 |
| O | 0.303408 | -1.731078 | 1.143885 |
| H | 1.885974 | -0.312446 | 2.654008 |
| O | 2.683302 | 2.220583 | 2.339086 |
| H | 1.659495 | 3.235935 | 0.227579 |
| H | 0.143036 | 2.082016 | -1.339037 |
| C | -0.729608 | -0.460078 | -1.143406 |
| S | -1.460707 | 0.147620 | -2.606674 |
| C | -2.233655 | -1.433165 | -2.820643 |
| N | -1.946708 | -2.285613 | -1.894765 |
| N | -1.118811 | -1.730524 | -0.973297 |
| C | -3.117698 | -1.748558 | -3.989353 |
| C | -2.454461 | -1.438476 | -5.317358 |
| H | -3.345049 | -2.812973 | -3.925398 |
| H | -4.063873 | -1.214150 | -3.879624 |
| C | -2.551827 | -0.143244 | -5.913602 |
| C | -1.844612 | 0.106473 | -7.135767 |
| C | -1.083720 | -0.933743 | -7.728096 |
| C | -1.018911 | -2.172324 | -7.140167 |
| C | -1.704676 | -2.418207 | -5.930493 |
| C | -3.311996 | 0.918622 | -5.351926 |
| C | -1.920221 | 1.392468 | -7.731116 |
| H | -0.555371 | -0.732172 | -8.653203 |
| H | -0.440332 | -2.966698 | -7.596252 |
| H | -1.635045 | -3.398419 | -5.472092 |
| C | -3.361999 | 2.154464 | -5.953114 |
| C | -2.657595 | 2.397627 | -7.153627 |
| H | -1.380786 | 1.567563 | -8.655420 |
| H | -2.705542 | 3.375848 | -7.617063 |
| H | -3.948972 | 2.947382 | -5.504949 |
| H | -3.870432 | 0.761650 | -4.438368 |
| H | 2.968964 | 1.671116 | 3.079826 |
| H | -0.747055 | -2.219234 | -0.135965 |

*trans*-enol 1

Total energy = -1390.983587 au

| | | | |
|---|---|---|---|
| C | 0.117376 | 0.179928 | -0.211699 |
| C | 0.011250 | 0.125883 | 1.157507 |
| C | 1.167810 | 0.048755 | 1.976228 |
| C | 2.460234 | 0.029525 | 1.360071 |
| C | 2.532705 | 0.088152 | -0.057325 |
| C | 1.391745 | 0.161872 | -0.822533 |
| C | 1.068238 | -0.010633 | 3.391277 |
| C | 2.197940 | -0.085686 | 4.166138 |
| C | 3.474827 | -0.107567 | 3.559166 |
| C | 3.621541 | -0.052914 | 2.190811 |
| C | 5.005127 | -0.112454 | 1.580454 |
| C | 5.265390 | -1.383835 | 0.825460 |
| S | 4.737174 | -2.941563 | 1.405783 |
| C | 5.474521 | -3.626365 | -0.036953 |
| N | 6.020050 | -2.709650 | -0.800571 |
| N | 5.895758 | -1.439463 | -0.311214 |
| C | 5.490488 | -5.045114 | -0.386532 |
| C | 4.922630 | -6.049056 | 0.418923 |
| C | 4.965942 | -7.389512 | 0.036553 |
| C | 5.578282 | -7.749647 | -1.160112 |
| C | 6.151957 | -6.771360 | -1.981121 |
| C | 6.101407 | -5.448178 | -1.589095 |
| O | 4.329739 | -5.670691 | 1.583575 |
| O | 5.648159 | -9.041319 | -1.582351 |
| H | 4.520702 | -8.144798 | 0.675655 |
| H | 6.625242 | -7.063704 | -2.909349 |
| H | 6.540805 | -4.684706 | -2.216230 |
| H | 5.747742 | -0.018716 | 2.377349 |
| H | 5.176051 | 0.715708 | 0.888274 |
| H | 0.084375 | 0.004146 | 3.847022 |
| H | 2.120359 | -0.130508 | 5.245996 |
| H | 4.357519 | -0.173171 | 4.185598 |
| H | -0.962509 | 0.140523 | 1.634746 |
| H | -0.773181 | 0.237261 | -0.826462 |
| H | 1.469053 | 0.205722 | -1.902632 |
| H | 3.497414 | 0.071115 | -0.546489 |
| H | 3.976117 | -6.435434 | 2.053975 |
| H | 5.222704 | -9.628440 | -0.945536 |

*trans*-enol 2

Total energy = -1390.981909 au

| | | | |
|---|---|---|---|
| C | 0.325217 | -0.358847 | 0.523278 |
| C | -0.047681 | -1.080479 | 1.672992 |
| C | 0.845377 | -1.357240 | 2.689355 |
| C | 2.166378 | -0.906512 | 2.579681 |
| C | 2.571293 | -0.187144 | 1.459145 |
| C | 1.658813 | 0.081358 | 0.439199 |

| | | | |
|---|---|---|---|
| H | -1.070201 | -1.424477 | 1.747129 |
| H | 0.542654 | -1.915372 | 3.565669 |
| O | 3.015367 | -1.200596 | 3.601666 |
| H | 3.592932 | 0.168199 | 1.374424 |
| O | 2.028168 | 0.778687 | -0.669127 |
| C | -0.673105 | -0.106006 | -0.513352 |
| S | -0.381344 | 0.760473 | -2.016317 |
| C | -2.061078 | 0.478423 | -2.401875 |
| N | -2.695750 | -0.189489 | -1.481472 |
| N | -1.913358 | -0.518773 | -0.410201 |
| C | -2.711942 | 0.935055 | -3.676429 |
| H | 3.897545 | -0.854678 | 3.418559 |
| C | -2.006500 | 0.415335 | -4.914081 |
| H | -3.734947 | 0.558136 | -3.651213 |
| H | -2.775486 | 2.025501 | -3.686435 |
| C | -2.397177 | -0.794759 | -5.445011 |
| C | -1.745427 | -1.354759 | -6.565828 |
| C | -0.693134 | -0.695562 | -7.150846 |
| C | -0.255570 | 0.554644 | -6.643096 |
| C | -0.921507 | 1.129595 | -5.510799 |
| H | -3.221183 | -1.332302 | -4.988841 |
| H | -2.081246 | -2.307837 | -6.957032 |
| H | -0.182205 | -1.119250 | -8.008272 |
| C | 0.831303 | 1.249071 | -7.235748 |
| C | -0.461427 | 2.386480 | -5.032023 |
| C | 0.596232 | 3.032578 | -5.627864 |
| C | 1.252881 | 2.459116 | -6.740177 |
| H | 1.325977 | 0.803076 | -8.091656 |
| H | 2.085182 | 2.978281 | -7.200550 |
| H | -0.947278 | 2.852684 | -4.184971 |
| H | 0.928003 | 3.989908 | -5.243462 |
| H | 2.963040 | 1.014677 | -0.633717 |

*trans*-enol 3

Total energy = -1390.980954 au

| | | | |
|---|---|---|---|
| C | 0.387939 | 0.327506 | -0.299205 |
| C | 0.464491 | 1.390047 | 0.621045 |
| C | 1.484060 | 1.488663 | 1.547280 |
| C | 2.477374 | 0.502411 | 1.577983 |
| C | 2.432417 | -0.565556 | 0.686664 |
| C | 1.396866 | -0.651183 | -0.243448 |
| H | -0.306603 | 2.147396 | 0.587399 |
| H | 1.529021 | 2.313767 | 2.246040 |
| O | 3.466477 | 0.638842 | 2.502566 |
| H | 3.198343 | -1.333426 | 0.709358 |
| O | 1.329326 | -1.683786 | -1.127071 |
| C | -0.715793 | 0.292883 | -1.255880 |
| S | -0.952199 | -0.930636 | -2.496929 |
| C | -2.382528 | -0.049200 | -2.970033 |
| N | -2.598885 | 1.009540 | -2.246991 |
| N | -1.656719 | 1.206310 | -1.276725 |
| C | -3.242294 | -0.484362 | -4.126157 |
| H | 4.100772 | -0.084960 | 2.430298 |
| C | -4.673417 | -0.010359 | -4.025509 |
| H | -2.798106 | -0.097046 | -5.047393 |
| H | -3.202787 | -1.574311 | -4.206585 |
| C | -5.138590 | 0.952449 | -4.892330 |
| C | -6.471855 | 1.419522 | -4.830215 |
| C | -7.336893 | 0.913357 | -3.893201 |
| C | -6.904654 | -0.083877 | -2.979831 |
| C | -5.554618 | -0.557017 | -3.041630 |
| H | -4.467925 | 1.365362 | -5.637723 |
| H | -6.803038 | 2.180060 | -5.527507 |
| H | -8.360904 | 1.265765 | -3.837033 |
| C | -7.784194 | -0.623740 | -2.005278 |
| C | -5.148032 | -1.553789 | -2.115164 |
| C | -6.024941 | -2.057437 | -1.182781 |
| C | -7.357270 | -1.588704 | -1.124843 |
| H | -8.804695 | -0.258355 | -1.968236 |
| H | -8.038139 | -1.992596 | -0.384811 |
| H | -4.131787 | -1.924651 | -2.136614 |
| H | -5.693177 | -2.818037 | -0.485665 |
| H | 2.067747 | -2.292768 | -1.004382 |

## References


[1] R. F. Pasternack, P. J. Collings, *Science* **1995**, *269*, 935-939.

[2] G. Jones II, W. R. Jackson, Ch. Y. Choi, W. R. Bergmark, *J. Phys. Chem.* **1985**, *89*, 294-300.

[3] R. R. Valiev, V. N. Cherepanov, G. V. Baryshnikov, D. Sundholm, *Phys. Chem. Chem. Phys.* **2018**, *20*, 6121-6133.

[4] S. Dhar, D. K. Rana, S. Singha Roy, S. Roy, S. Bhattacharya, S. C. Bhattacharya, *J. Lumin.* **2012**, *132*, 957-964.

[5] Gaussian 16, Revision C.01, M. J. Frisch, G. W. Trucks, H. B. Schlegel, G. E. Scuseria, M. A. Robb, J. R. Cheeseman, G. Scalmani, V. Barone, G. A. Petersson, H. Nakatsuji, X. Li, M. Caricato, A. V. Marenich, J. Bloino, B. G. Janesko, R. Gomperts, B. Mennucci, H. P. Hratchian, J. V. Ortiz, A. F. Izmaylov, J. L. Sonnenberg, D. Williams-Young, F. Ding, F. Lipparini, F. Egidi, J. Goings, B. Peng, A. Petrone, T. Henderson, D. Ranasinghe, V. G. Zakrzewski, J. Gao, N. Rega, G. Zheng, W. Liang, M. Hada, M. Ehara, K. Toyota, R. Fukuda, J. Hasegawa, M. Ishida, T. Nakajima, Y. Honda, O. Kitao, H. Nakai, T. Vreven, K. Throssell, J. A. Montgomery, Jr., J. E. Peralta, F. Ogliaro, M. J. Bearpark, J. J. Heyd, E. N. Brothers, K. N. Kudin, V. N. Staroverov, T. A. Keith, R. Kobayashi, J. Normand, K. Raghavachari, A. P. Rendell, J. C. Burant, S. S. Iyengar, J. Tomasi, M. Cossi, J. M. Millam, M. Klene, C. Adamo, R. Cammi, J. W. Ochterski, R. L. Martin, K. Morokuma, O. Farkas, J. B. Foresman, and D. J. Fox, Gaussian, Inc., Wallingford CT, 2016.

[6] J. Tomasi, B. Mennucci, R. Cammi, *Chem. Rev.* **2005**, *105*, 2999-3094.

[7] M. Cossi, V. Barone, R. Cammi, J. Tomasi, *Chem. Phys. Lett.* **1996**, *255*, 327-335.

[8] G. Scalmani, M. J. Frisch, *J. Chem. Phys.* **2010**, *132*, 114110.

[9] M. Cossi, V. Barone, *J. Chem. Phys.* **2001**, *115*, 4708-4717.

[10] G. Scalmani, M. J. Frisch, B. Mennucci, J. Tomasi, R. Cammi, V. Barone, *J. Chem. Phys.* **2006**, *124*, 094107.

[11] A. D. Becke, *J. Chem. Phys.* **1993**, *98*, 5648-5652.

[12] C. Lee, W. Yang, R. G. Parr, *Phys. Rev. B,* **1988**, *37*, 785-789.

[13] P. J. Stephens, F. J. Devlin, C. F. Chabalowski, M. J. Frisch, *J. Phys. Chem.* **1994**, *98*, 11623-11627.

[14] R. Krishnan, J. S. Binkley, R. Seeger, J. A. Pople, *J. Chem. Phys.* **1980**, *72*, 650-654.

[15] T. Clark, J. Chandrasekhar, G. W. Spitznagel, P. Von Ragué Schleyer, *J. Comput. Chem.* **1983**, *4*, 294-301.

[16] S. Grimme, J. Antony, S. Ehrlich, H. Krieg, *J. Chem. Phys.* **2010**, *132*, 154104.

[17] S. Grimme, S. Ehrlich, L. Goerigk, *J. Comput. Chem.* **2011**, *32*, 1456-1465.

[18] T. H. Dunning Jr., *J. Chem. Phys.* **1989**, *90*, 1007-1023.

[19] R. A. Kendall, T. H. Dunning Jr., R. J. Harrison, *J. Chem. Phys.* **1992**, *96*, 6796-6806.

[20] GaussView, Version 5.0.9, R. Dennington, T. A. Keith, J. M. Millam, Semichem Inc., Shawnee Mission, KS, 2009.

[21] A. Matwijczuk, D. Karcz, K. Pustuła, M. Makowski, A. Górecki, D. Kluczyk, M. M. Karpińska, A. Niewiadomy, M. Gagoś, *J. Lumin.* **2018**, *201*, 44-56.

[22] A. Matwijczuk, A. Górecki, M. Makowski, K. Pustuła, A. Skrzypek, J. Waś, A. Niewiadomy, M. Gagoś, *J. Fluoresc.* **2018**, *28*, 65-77.

[23] I. Budziak, D. Karcz, M. Makowski, K. Rachwał, K. Starzak, A. Matwijczuk, B. Myśliwa-Kurdziel, A. Oniszczuk, M. Combrzyński, A. Podleśna, A. Matwijczuk, *Int. J. Mol. Sci.* **2019**, *20*, 5494.

[24] I. Budziak, D. Karcz, M. Makowski, B. Myśliwa-Kurdziel, K. Kasprzak, A. Matwijczuk, E. Chruściel, A. Oniszczuk, L. Adwent, A. Matwijczuk, *J. Mol. Liq.* **2019**, *291*, 111261.

[25] G. Czernel, I. Budziak, A. Oniszczuk, D. Karcz, K. Pustuła, A. Górecki, A. Matwijczuk, B. Gładyszewska, M. Gagoś, A. Niewiadomy, A. Matwijczuk, *Molecules* **2020**, *25*, 4168.

[26] A. Kudelko, M. Olesiejuk, M. Luczynski, M. Swiatkowski, T. Sieranski, R. Kruszynski, *Molecules* **2020**, *25*, 2822.

[27] R. D. Ila, S. P. Verma, G. Krishnamoorthy, *Photochem. Photobiol. Sci.* **2020**, *19*, 844-853.
